# Readmission and survival of hospitalized pulmonary tuberculosis patients: a nationwide record-based cohort analysis in Thailand (2017–2022)

**DOI:** 10.1186/s40249-026-01467-0

**Published:** 2026-06-15

**Authors:** Kyaw Ko Ko Htet, Nyi Nyi Zayar, Satiti Palupi, Rassamee Chotipanvithayakul, Vorthunju Nakhonsri, Chumpol Ngamphiw, Peerapat Khunkham, Sanya Vasoppakarn, Narumol Atthakul, Sissades Tongsima, Chantisa Keeratipusana, Watcharapot Janpoung, Ponlagrit Kumwichar, Virasakdi Chongsuvivatwong

**Affiliations:** 1 Department of Epidemiology, Faculty of Medicine, Prince of Songkla University, Hat Yai, Songkhla, Thailand; 2 Department of Communicable Disease, East Java Provincial Health Office, Surabaya, East Java Indonesia; 3 National Biobank of Thailand, National Science and Technology Development Agency, Khlong Luang, Pathum Thani, Thailand; 4 Bureau of Service Quality Development, National Health Security Office, Lak Si, Bangkok, Thailand

**Keywords:** Tuberculosis, Survivor, Readmission, Thailand

## Abstract

**Background:**

Assessing the readmitted diseases and survivors in patients hospitalized with pulmonary tuberculosis (TB) is crucial for healthcare strategies tailored to TB survivors. This study aims to compare readmissions and survival of hospitalized pulmonary TB patients with those of their matched controls in Thailand.

**Methods:**

We retrieved inpatient drug-sensitive pulmonary TB cases from the Thai Health Information Portal database (2017–2022), which included all hospitalized admissions in Thai hospitals. Non-TB controls were selected using 1∶1 propensity score matching by admission date, location, sex, age, and comorbidities. Time to readmission and to death event were the outcome variables. Cox regression with a robust variance sandwich estimator was used and then the E-value was computed. E-values indicate the strength of possible unexplained confounders.

**Results:**

From the propensity score matching, a total of 59,027 patients newly admitted for TB could be matched with 59,027 non-TB inpatient controls. Hazard ratios (HR) with 95% confidence intervals (*CI*) and E-values for causes of readmission among TB patients were 5.4 (2.1–14.3) and 10.2 for pericarditis, 5.4 (4.1–7.2) and 10.2 for pneumothorax, 3.8 (2.9–4.9) and 7.1 for bronchiectasis, 3.1 (2.4–4.2) and 5.6 for hyperfunction of the pituitary gland, and 2.4 (2.1–2.8) and 4.2 for inflammatory liver diseases. The TB cohort also had a higher risk of death [HR = 1.5 (95% *CI: *1.4–1.6) and E-value = 2.3].

**Conclusions:**

Hospitalized TB patients in Thailand are at higher risk than their non-TB controls in getting readmissions for thoracic problems and death. These problems call for improvement of TB care.

**Graphical Abstract:**

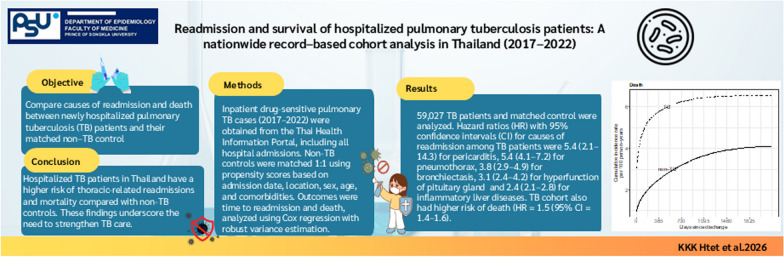

**Supplementary Information:**

The online version contains supplementary material available at 10.1186/s40249-026-01467-0.

## Background

Global tuberculosis control efforts are estimated to have saved 83 million lives between 2000 and 2024 [1]. Although most survivors of pulmonary TB are cured, more than half of the patients with TB diseases are readmitted to health care services for persistent and long-term health problem [2]. In addition, the increase in TB-related deaths primarily occurred in the highest TB burden countries, especially in Southeast Asia (SEA) [1].

Known burden of pulmonary TB involves the functional impairment of lungs and other organs [3, 4]. Without this awareness, the health impact attributable to TB would be undervalued [1]. A systematic review has highlighted the significant readmissions with abnormal lung function, persistent respiratory symptoms, and radiological abnormalities among TB cohort patients in SEA countries [5]. Nonetheless, there have been limited studies evaluating readmitted diseases and survival of the so-called “cured TB”.

Thailand is one of the high-TB-burden countries in SEA [1]. There is no official register for “readmitted diseases among TB cohort patients” in the TB control program. The Thai Hospital Information Portal (THIP) is a database from the data warehouse of National Health Security Office (NHSO), Thailand. The database covers all hospitalized admissions which include TB at public hospitals where individual electronic records are sent to the NHSO [6].

The objective of the study was to compare the TB and non-TB hospitalized patients on their subsequent admissions and death. The information would be useful for planning of TB care in low-and middle-ncome (LMIC) countries.

## Methods

### Data source

The admission records of inpatient pulmonary TB and non-TB patients were retrieved from THIP database for a period of 2016 to 2022. The THIP database is from a collaborative data warehouse project managed jointly by the NHSO, the Institute of Research and Development for Health of Southern Thailand, the National Science and Technology Development Agency, and Prince of Songkla University, Thailand [6]. The database was accessed on 10 January 2023. With unique citizen identification number, the records are systematically encrypted, but it is still possible to link the admission records of the same patients. By these means, it was possible to see how TB patients could be admitted at one time and return for subsequent admissions for different reasons.

The collected data include comprehensive information without any missing entries, encompassing patient codes, age, sex, dates and times of hospital admission, super-district areas, principal diagnosis, and secondary diagnoses (up to 20 items) which are coded using the International Classification of Diseases, Tenth Revision (ICD-10) system, and data on death from hospital records and the national death register.

### Study design

A retrospective cohort analysis was conducted using the de-identified, encrypted identification numbers. Two cohorts were formed, consisting of matched pairs of hospitalized pulmonary TB and non-TB patients. The outcomes were subsequent admissions and mortality. We also further examined these outcomes among TB patients for whom we could not find a suitable matched control.

### Pulmonary TB patients

Pulmonary TB cohort comprised the newly admitted pulmonary TB patients, with primary diagnoses of bacteriologically confirmed TB and clinically diagnosed TB, indicated by ICD-10 codes of A15 and A16, respectively. These patients were hospitalized for the first time due to TB, even if the diagnosis was established earlier. Patients < 15 years, those admitted before 2017, and history of drug resistance (ICD-10 codes of U88) in any admission were excluded from the TB group. TB cases without pulmonary involvement were not used because these small minorities have different affected organs.

### Non-TB control cohort

Potential non-TB control cohort were selected from all patients who had admission for any conditions except ICD-10 codes of A15 and A16. The control patients with the same day of hospitalization, an age interval of 5 years, sex, and super-district area, as TB cases were initially retrieved from the THIP database and used only once. As more than one potential control was available for each case, the most appropriate control was selected using propensity score matching.

### Propensity score matching

Among TB cases and above-mentioned potential control pool, the propensity score was calculated using logistic regression for the predicted probability of having TB and non-TB based on covariates of age, sex, and histories of HIV, diabetes mellitus, chronic obstructive pulmonary disease, any type of cancer, heart failure and ischemic heart disease [7]. A 1∶1 propensity score matching was carried out with non-TB controls using the nearest neighbor method with a caliper width set at 0.2 of the standard deviation of the logit of propensity score using matchit R package [8]. The propensity score matching filtered out the TB patients who had no matched non-TB control [10]. This group was analyzed separately to explore the distribution of the outcome variables without any attempt for comparison with matched TB and non-TB groups.

### Outcome variables

The readmission rate, length of hospital stay, death from hospital record and national death register and cause of readmissions were outcome variables. Death data were ascertained from hospital records (for in-hospital mortality) and from the national death registry, which are routinely updated in the THIP database. Cause of death was verified only in hospital records..

We examined a broad range of causes of readmission to allow us to understand the disease nature. They included cardiovascular (ICD-10 codes of I00–I99) and pulmonary diseases (J00–J99) and extra-thoracic organs including endocrine (E00–E35), central nervous system (G00–G99), digestive system (K00–K95), musculoskeletal system (M00–M99), and genitourinary system (N00–N99).

### Data management and analysis

The data were managed and analyzed using R version 4.2.2 (The R Foundation for Statistical Computing, Vienna, Austria) [9].

The mean propensity score of covariates was compared using a student’s* t*-test between pre- and post-matched TB and non-TB groups. A non-significant result (*P* > 0.05) indicated that covariates were comparable between two matched groups. Distribution of clinical covariates among matched groups was presented as number and percentage.

In time-to-event analysis, time zero was defined as the discharge date from index TB and non-TB admissions. Failure event took place if the subject either died or was readmitted in the first time. Censored records included patients who survived without readmission until the end of follow-up, patients who died outside the hospital without a recorded readmission, and those for whom the study ended before the failure event occurred. All survival patients were followed up in the record until September 2022 to calculate person-year of follow**-**up.

Total readmissions and death per 100 person-year of follow-up were calculated for matched groups. Length of hospital stay was calculated by days from admission and discharge date among readmitted patients. Average length of hospital stay per readmission was computed for matched groups.

Survival time was calculated for each interest of event including causes of readmission and death between matched groups. Incidence of 10,000 person-year at risk was calculated using the following formula:

$$\text{Incidence of interest of event per 10,000 person-years at risk} =\frac{\text{Number of interest of event}}{\text{Sum of follow-up years (per 10,000 person-year)}}$$

The stratified log-rank test was used to compare the survival distribution of the events of interests between matched groups [10]. Events of interests with significantly different distribution between matched TB and non-TB from stratified log-rank test were furthered analyzed using Cox regression.

A Cox regression with a robust variance sandwich estimator was used to estimate the hazard ratio (HR) with 95% confidence interval (*CI*) [10]. The estimate of HR > 1 was interpreted as the relative increase in the hazard of event of interests occurring in matched TB compared with non-TB [10]. The significance level was set at a two-sided *P*-value of less than 0.05.

Despite our above attempt to control confounders by propensity score matching and regression, this study might not be free from other unmeasured confounders. We therefore did further analysis to quantify the degree of the possible confounders missing in the model. We computed E-value [11]. A higher E-value indicates that an unmeasured confounder would need to have a stronger association with both the exposure and the outcome, greater than the magnitude specified by the E-value, to fully explain away the observed association. Therefore, larger E-values suggest that the observed associations are less likely to be explained away by unmeasured confounders. Additionally, a post-hoc power calculation was performed to determine whether the study had sufficient power to detect differences in the survival curves of outcomes between matched TB and non-TB groups under the Cox proportional hazards model. A power close to 1 would indicate adequate power [12].

Finally, for the TB group without matched controls, the outcomes—i.e., causes of readmissions and deaths—were presented as incidence rates per person–years to explore the distribution of outcomes.

## Results

### Dataset of TB and non–TB patients

**Fig.1:**
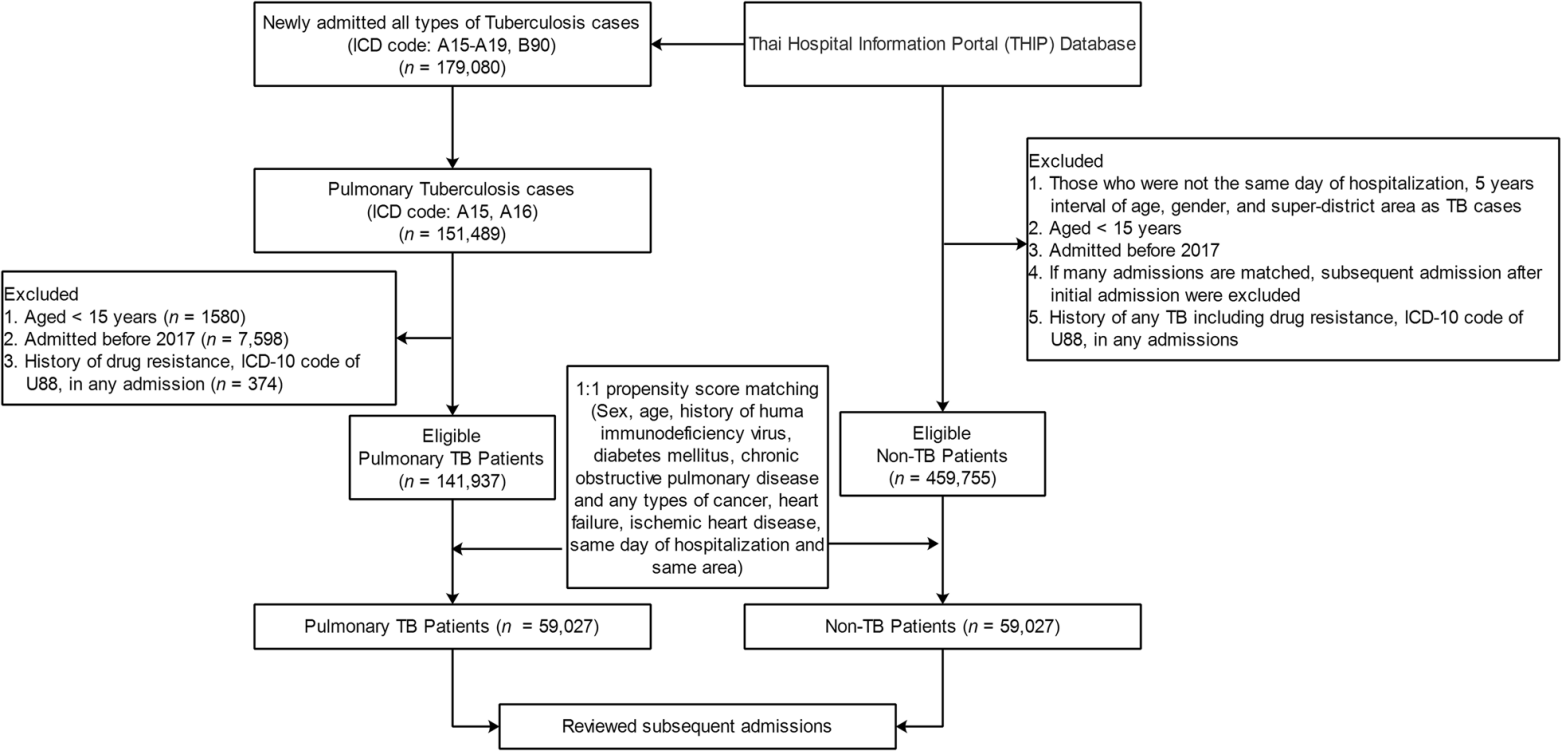
Flowchart presentation on the selection of matched non-tuberculosis, tuberculosis and unmatched tuberculosis patients. *TB* tuberculosis

The selection of patients among pulmonary TB and non-TB groups is shown in Fig. 1. For the whole database, 179,080 records had all kinds of TB as the primary diagnosis. Among them, 151,489 patients were experiencing their first admission of pulmonary TB. After removing 9552 patients that met the exclusion criteria, 141,937 patients were eligible for the analysis.

A total of 459,755 eligible non-TB patients were selected for 1∶1 propensity score matching with the TB cases. Finally, 59,027 matched TB-non-TB patient sets were available and used for the analysis, while 82,910 TB patients had no match with non-TB.

**Fig. 2:**
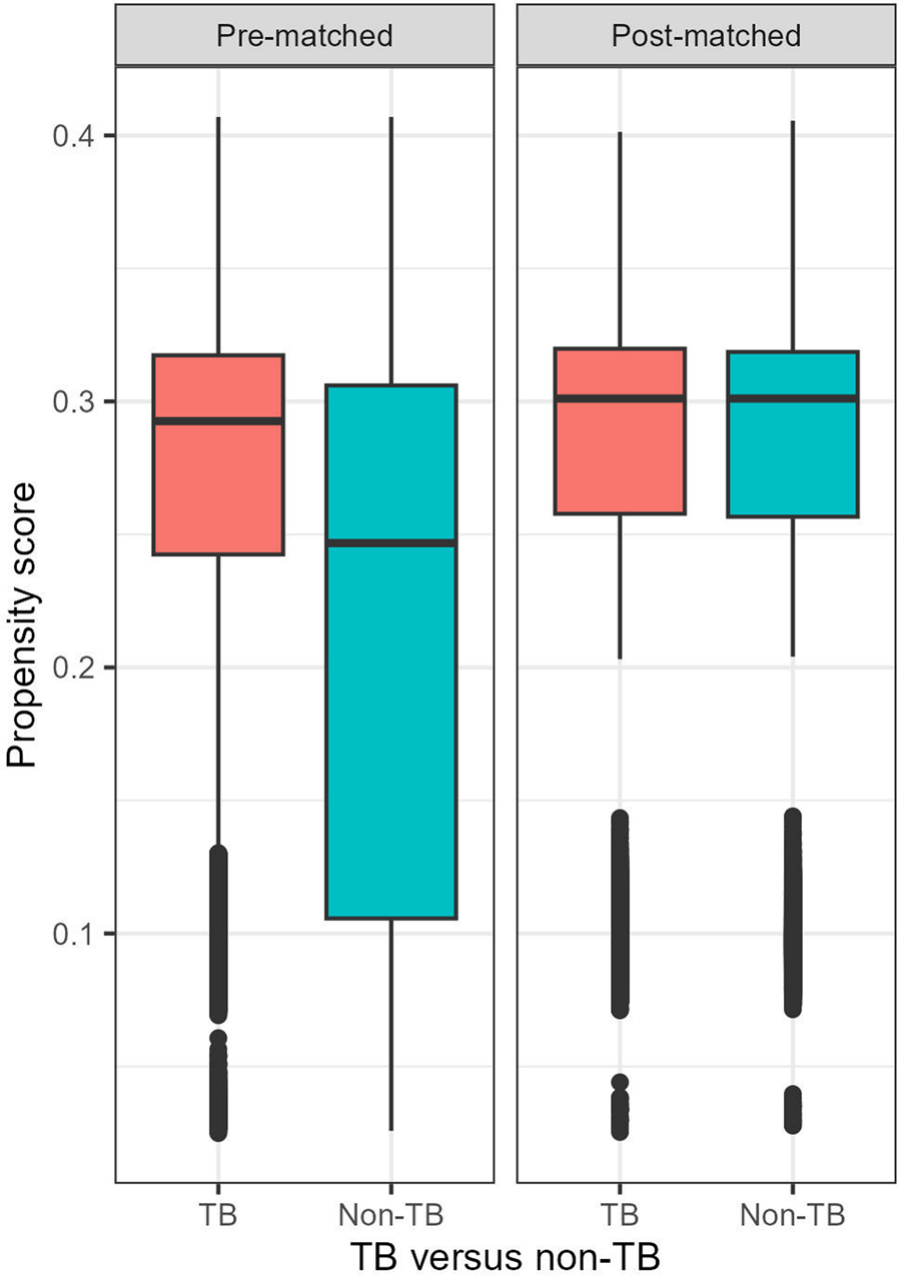
Distribution of propensity score for covariates in tuberculosis (TB) and non-TB before and after propensity score matching in Thailand

Figure 2 shows that the average propensity score was higher in the TB group than in the non-TB group before matching, indicating that the covariate distribution was significantly different between the two groups (*P* < 0.001). In contrast, the average propensity score was approximately 0.3 in both groups after matching, suggesting improved comparability of covariates between the groups (*P* = 0.7).

### Background characteristics of TB and their matched non-TB

In Table 1, of 141,937 TB cases identified in the databases, only 59,027 (41.6%) could be matched with non**-**TB controls with a close propensity score. The sex and age distribution of the matchable and unmatchable TB cases was close. However, the matchable TB cases were less likely to have comorbidities such as human immunodeficiency virus, diabetes mellitus, chronic obstructive pulmonary disease, any types of cancer, heart failure and ischemic heart disease. Eventually, we obtained matched sets which were predominated by males, with a mean age of 57 years, a history of ischemic heart disease and diabetes mellitus in over 20% of the cases, and a 1–5% prevalence of other major comorbidities such as human immunodeficiency virus (0.7%), heart failure (2.9%), chronic obstructive pulmonary disease (3.1%), and cancer (4.8%).

**Table 1:** Distribution of hospitalized patients in TB and their matched non-TB cohorts in Thailand (2017–2022)

Background characteristics	Post-matched		Unmatched
	Non-TB	TB	TB
Female, *n* (%)	16,201 (27.4)	16,184 (27.4)	23,304 (28.1)
Age (years), Mean (Standard deviation)	57.1 (15.2)	57.0 (15.6)	57.2 (17.8)
History of human immunodeficiency virus, *n* (%)	418 (0.7)	578 (1.0)	3566 (4.3)
History of diabetes mellitus, *n* (%)	12,407 (21.0)	12,701 (21.5)	26,547 (32.0)
History of chronic obstructive pulmonary disease, *n* (%)	1817 (3.1)	2169 (3.7)	7803 (9.4)
History of any types of cancer, *n* (%)	2809 (4.8)	2595 (4.4)	5819 (7.0)
History of heart failure, *n* (%)	1688 (2.9)	1902 (3.2)	5265 (6.4)
History of ischemic heart disease, *n* (%)	16,247 (27.5)	16,446 (27.9)	40,428 (48.8)

*TB* Tuberculosis; Exact match was done for same day of hospitalization and super-district areas

Lastly, among the remaining unmatched TB cases, the most common comorbidities were diabetes mellitus (32.0%) and ischemic heart disease (48.8%), while other comorbidities ranged from 4 to 10%.

Supplementary Table 1 shows the distribution of primary ICD-10 diagnoses of matched non-TB patients. Five common ICD-10 diseases of non-TB control accounted for 58.3% of the control.

### Readmission, length of hospital stays and death of TB, and their matched non-TB cohorts

As shown in Table 2, based on 59,027 matched sets, TB patients had a lower readmission rate (14.9 versus 30.3 per 100 person**-**year) but a longer length of hospital stay per readmission (5.0versus 3.5 days), a large proportion of in-hospital mortality (10.8% versus 5.6% of all patients), and a slightly higher total mortality rate per 100 person-years of follow-up (6.5 versus 4.0 per 100 person**-**year, respectively). Out of total in-hospital mortality among TB and non-TB patients, the proportions that occurred during the first admission was 4511 out of 6382 (70.6%) and 1659 out of 3302 (50.2%), respectively.

**Table 2:** Readmission, length of hospital stays and death of TB and their matched non-TB cohorts

Readmission, length of stay and death	Matched		Unmatched
	Non-TB	TB	TB
Total patients, *n*	59,027	59,027	82,910
Total readmission, *n*	53,532	24,420	39,947
Total length of stay (days), *n*	267,315	169,665	274,181
Total death, *n*	7221	10,699	15,055
Total person-year of follow-up, *n*	176,693	163,351	222,673
Readmission			
Readmission rate per 100 person-year of follow-up, *n* (95% CI)	30.3 (30.0–30.5)	14.9 (14.7–15.1)	17.9 (17.7–18.1)
Length of stay (days) per readmission, Median (Interquartile range)	3.5 (2.0–6.3)	5.0 (2.5–8.5)	5.0 (2.8–8.5)
Death			
No death, *n* (%)	51,806 (87.8)	48,328 (81.9)	67,855 (81.8)
In-hospital mortality, *n* (%)	3302 (5.6)	6382 (10.8)	822 (9.9)
Death from national death register, *n* (%)	3919 (6.6)	4317 (7.3)	6834 (8.2)
Death per 100 person-year of follow-up, *n* (95% CI)	4.0 (3.9–4.1)	6.5 (6.4–6.7)	6.7 (6.6–6.8)

The unmatched TB cases in the last column showed similar figures to the matched TB cases. Among the 82,910 unmatched TB cases, their readmission rate was 17.9 per 100 person-years. The unmatched group also had a longer length of stay (5.0 days per readmission), as well as higher in-hospital mortality (9.9% of all patients) and total death rates (6.7 per 100 person-years of follow-up) than the matched non**-**TB group.

### Comparison of matched TB and non-TB cohorts on common readmissions with thoracic, and extra-thoracic diseases

As shown in Table 3, TB and non-TB cohorts were compared in terms of the most common readmissions: 10 cardiovascular, 11 pulmonary, and 12 extra-thoracic diseases. Readmissions among matched TB cases were ordered in decreasing frequency according to thoracic and extra-thoracic diseases.

**Table 3:** Comparison of matched TB and non-TB cohorts on common readmissions with thoracic, and extra-thoracic diseases in Thailand (2017–2022)

ICD-10	Thoracic, and extra-thoracic diseases events	Matched	
		Non-TB	TB
Total patients, *n*		59,027	59,027
Thoracic diseases			
Cardiovascular diseases			
I10	Essential (primary) hypertension, *n* (%)	8120 (13.8)	2984 (5.1)
I48	Atrial fibrillation and flutter, *n* (%)	1066 (1.8)	437 (0.7)
I50	Heart failure, *n* (%)	736 (1.2)	382 (0.6)
I25	Chronic ischemic heart disease, *n* (%)	1289 (2.2)	353 (0.6)
I69	Sequelae of cerebrovascular disease, *n* (%)	1257 (2.1)	287 (0.5)
I95	Hypotension, *n* (%)	300 (0.5)	272 (0.5)
I21	Acute myocardial infarction, *n* (%)	544 (0.9)	183 (0.3)
I63	Cerebral infarction, *n* (%)	869 (1.5)	179 (0.3)
I47	Paroxysmal tachycardia, *n* (%)	214 (0.4)	147 (0.2)
I32	Pericarditis, *n* (%)	5 (0.001)	28 (0.05)
Pulmonary diseases			
J96	Respiratory failure, *n* (%)	1804 (3.1)	1983 (3.4)
J15	Bacterial pneumonia, *n* (%)	894 (1.5)	1450 (2.5)
J90	Pleural effusion, *n* (%)	346 (0.6)	594 (1)
J93	Pneumothorax, *n* (%)	61 (0.1)	334 (0.6)
J47	Bronchiectasis, *n* (%)	71 (0.1)	272 (0.5)
J12	Viral pneumonia, *n* (%)	365 (0.6)	148 (0.3)
J45	Asthma, *n* (%)	347 (0.6)	118 (0.2)
J20	Acute bronchitis, *n* (%)	281 (0.5)	111 (0.2)
J86	Pyothorax, *n* (%)	61 (0.1)	108 (0.2)
J69	Pneumonitis due to solids and liquids, *n* (%)	242 (0.4)	105 (0.2)
J85	Abscess of lung and mediastinum, *n* (%)	39 (0.1)	85 (0.1)
Extra–thoracic diseases			
E83	Disorders of mineral metabolism, *n* (%)	2362 (4)	1834 (3.1)
N18	Chronic kidney disease, *n* (%)	3974 (6.7)	1495 (2.5)
N17	Acute kidney failure, *n* (%)	2215 (3.8)	1403 (2.4)
E78	Disorders of lipoprotein metabolism and other lipidemias, *n* (%)	3920 (6.6)	1069 (1.8)
M10	Arthropathy, *n* (%)	1336 (2.3)	760 (1.3)
N39	Other disorders of urinary system, *n* (%)	1799 (3.0)	687 (1.2)
K92	Other diseases of digestive system, *n* (%)	1012 (1.7)	609 (1.0)
K75	Inflammatory liver diseases, *n* (%)	251 (0.4)	608 (1.0)
E16	Other disorders of pancreatic internal secretion, *n* (%)	540 (0.9)	512 (0.9)
K74	Fibrosis and cirrhosis of liver, *n* (%)	915 (1.6)	486 (0.8)
K70	Alcoholic liver disease, *n* (%)	723 (1.2)	374 (0.6)
E22	Hyperfunction of pituitary gland, *n* (%)	66 (0.1)	205 (0.3)

*ICD-10* International Classification of Diseases, Tenth Revision; *TB* Tuberculosis

All cardiovascular readmissions were less common in the TB group except for pericarditis (0.05% in TB versus 0.001% in non-TB). The same was true for extra-thoracic readmissions, with the exceptions of inflammatory liver diseases (1.0% in TB versus 0.4% in non-TB) and pituitary hyperfunction (0.3% in TB versus 0.1% in non-TB). The opposite was observed for pulmonary readmissions, which were more frequent except for viral pneumonia, asthma, acute bronchitis, and pneumonitis due to solids and liquids.

It should be noted that Table 3 does not present incidence density because the matched pairs had the same person-time at risk. Therefore, the count of events is presented for simpler interpretation.

In Supplementary Table 2, we have also included the unmatched TB groups. Overall, their incidence density for common readmission was similar to that of the matched TB patients.

Table 4 shows the list of diseases associated with readmissions with a significant HR of > 1 with their corresponding E-values (lower *CI*) for the associations between matched TB and non-TB. As expected, risk factors with larger HR correspondingly exhibited higher E-values. The items with an E-value > 4 included pericarditis (10.2), pneumothorax (10.2), bronchiectasis (7.1), hyperfunction of pituitary gland (5.6) and inflammatory liver diseases (4.2). The combination of death from the hospital record and from the national death register were significantly higher in the TB cohort than in the non-TB cohort (*P* < 0.001) although the E-value was relatively small(2.3).

**Table 4:** Hazard ratio of thoracic, extra-thoracic diseases and death among the hospitalized patients with matched TB and non-TB controls in Thailand (2017–2022)

ICD-10	Thoracic, extra-thoracic diseases and death events	Hazard ratio* of event in matched TB compared to non-TB (95% *CI*)	E-value (Lower* CI*)
Cardiovascular diseases			
I32	Pericarditis	5.4 (2.1–14.3)	10.2 (3.6)
Pulmonary diseases			
J93	Pneumothorax	5.4 (4.1–7.2)	10.2 (7.6)
J47	Bronchiectasis	3.8 (2.9–4.9)	7.1 (5.2)
J85	Abscess of lung and mediastinum	2.2 (1.5–3.2)	3.8 (2.3)
J90	Pleural effusion	1.7 (1.5–1.9)	2.7 (2.3)
J86	Pyothorax	1.7 (1.2–2.4)	2.7 (1.6)
J15	Bacterial pneumonia	1.6 (1.5–1.7)	2.5 (2.3)
J96	Respiratory failure	1.1 (1.03–1.2)	1.4 (1.2)
Extra–thoracic diseases			
E22	Hyperfunction of pituitary gland	3.1 (2.4–4.2)	5.6 (4.2)
K75	Inflammatory liver diseases	2.4 (2.1–2.8)	4.2 (3.6)
Death			
In-hospital mortality		1.9 (1.8–2.0)	3.2 (3.0)
Total Death (Death from hospital record and national death register)		1.5 (1.4–1.6)	2.3 (2.1)

95*% CI* 95% confidence interval; *ICD-10* International Classification of Diseases, Tenth Revision; *TB* Tuberculosis

*The values were adjusted for sex, age, history of human immunodeficiency virus, diabetes mellitus, chronic obstructive pulmonary disease, any type of cancer, heart failure and ischemic heart disease

In supplementary Table 3, the post-hoc power analysis results ranged from 0.83 to 1, indicating that the study had sufficient power to detect differences in the survival probability between two groups.

### Causes of death

As shown in Table 5, the cause-specific death were compared between the two matched cohorts. The most common cause of death in the TB cohort was certain infectious and parasitic diseases. There were 5459 deaths in the TB group compared with 194 in the control [HR (95% *CI*) = 29.6 (25.6–34.2)]. Of these, deaths due to TB numbered 5369, accounting for 84.1% of all deaths, compared with 0 death in the control, indicating that the TB group died significantly earlier than the control. The remaining causes of death were more common in the non-TB cohort.

**Table 5:** Comparison of hospitalized matched TB and non-TB cohort on hazard ratio of cause-specific death in Thailand (2017–2022)

ICD-10	Cause-specific death	Matched		Hazard ratio of death in matched TB compared to non-TB (95% *CI*)
		Non-TB	TB	
	Total (*n*)	3302	6382	
A00–A99	Certain infectious and parasitic diseases	194	5459	29.6 (25.6–34.2)
A15–A19	TB	0	5369	NA
J00–J99	Diseases of the respiratory system	781	462	0.62 (0.55–0.69)
I00–I99	Diseases of the circulatory system	656	90	0.14 (0.11–0.17)
K00–K99	Diseases of the digestive system	420	82	0.20 (0.16–0.25)
B00–C99	Neoplasms	274	80	0.30 (0.24–0.39)
Z00–Z99, R00–R99	Others	178	81	0.5 (0.3–0.6)
N00–N99	Diseases of the genitourinary system	289	65	0.23 (0.18–0.31)
G00–G99	Diseases of the nervous system	65	24	0.39 (0.24–0.62)
E00–E90	Endocrine, nutritional and metabolic diseases	59	22	0.39 (0.24–0.77)
S00–T98	Injury, poisoning and certain other consequences of external causes	291	11	0.03 (0.02–0.07)
M00–M99	Diseases of the musculoskeletal system and connective tissue	71	5	0.07 (0.02–0.18)
D50–D89	Diseases of the blood and blood-forming organs and certain disorders involving the immune mechanism	17	1	0.06 (0.08–0.46)
F00–F99	Mental, Behavioral and Neurodevelopmental disorders	6	1	0.17 (0.02–1.44)

*ICD-10* International Classification of Diseases, Tenth Revision; *TB* Tuberculosis; *NA* not allowed

In supplementary Table 4, we summarized the incidence of cause-specific death for the matched pairs and unmatched TB cases. The incidence of cause-specific death was similar between the matched and unmatched TB groups but different from that of the matched non-TB group.

## Discussion

Using the propensity score matching, we successfully compared 59,027 matched TB cases with 41,937 hospitalized TB patients and their non-TB counterparts. The TB cohort experienced about half the number of readmissions compared with the non-TB group. Readmissions in the TB cohort were more often complicated by thoracic diseases and needed longer hospital stays. The TB group also died earlier, with a 50% higher risk compared with the non-TB group. Similarly, the remaining unmatched TB cases showed similar results on causes of readmission and death.

In our study, the lower readmission rate with a higher death rate may reflect that TB patients might have delayed their decision to revisit the hospital until the disease became very severe. This delay might be explained by the fact that TB is a disease prevalent among people of lower socioeconomic status who have less social network support [13]. Stigmatization could also lead to delayed arrival at the hospital until the condition worsens [14]. Biologically, TB needing admission might also more severe damage than non-TB diseases, causing the initial survivors to worsen and succumb earlier.

Pulmonary damage was a significant cause of readmissions for thoracic diseases among TB patients. Structural pulmonary damage in TB can be explained by interactions between host immunity and infection as well as damage repair [2]. Even though TB patients who had their first admission could be discharged with improvement, a large proportion still suffered from residual damage requiring readmissions. Organs in close proximity to the lung were the most common targets. These conditions were also complicated by pneumonitis, pleural effusion, pneumothorax, bronchiectasis pyothorax, and abscess. Lung damage due to TB is a major, under-recognized public health challenge and a common cause of non-communicable respiratory disease in TB-endemic regions. Moving beyond the paradigm of "bacteriologically cure" at the end of TB therapy is essential [15].

Another complication of thoracic diseases among TB patients was pericarditis. TB infection is a leading cause of pericarditis in LMIC countries with high TB endemicity [16]. Early symptoms like fever and chest pain are often mistaken for a lung infection, leading to a missed diagnosis in about 20% of cases until the damage is permanent [17]. Unlike typical heart disease, tuberculous pericarditis attacks the heart's protective sac, the pericardium. While anti-TB drugs kill the bacteria, they cannot reverse the resulting scarring, leaving the heart trapped in a fibrosis casing even after the infection is cured [18]. In addition, a pulmonary TB focus may limit the occurrence of disseminated comorbidities.

In our study, pulmonary TB commonly involves inflammatory liver changes. Liver damage often persists after treatment completion, stemming from drug-induced injury, immune-mediated inflammation, or undetected hepatic diseases. Long-term monitoring reveals fibrosis or dysfunction in some cases, particularly in TB high-burden regions [19]. Pituitary hyperfunction was more common in the TB cohort. The mechanism of this phenomenon needs more extensive studies to explain [20].

Non-TB groups showed higher rates of extra-thoracic diseases such as mineral disorders, chronic kidney diseases, lipidemias, arthropathy, urinary/pancreatic issues, and liver diseases (inflammatory, fibrosis, alcoholic). This was due to a broader range of baseline primary hospitalized diseases, comorbidities in the male-dominated and elderly study population, and especially the complications of metabolic syndrome, which are common in LMICs nowadays [21].

The combined data from hospital and national death registers showed a higher rate in the TB cohort than that in non-TB cohort. We used propensity score matching to control for potential cofounders, including age, sex, immune status (human immunodeficiency virus and cancers), and chronic diseases such as diabetes mellitus, chronic obstructive pulmonary diseases, heart failure, and ischemic heart disease. However, we could not account for other potential confounders, such as smoking, alcohol consumption, low body mass index, and socioeconomic disadvantage. Additionally, disease severity and treatment outcomes may also impact the differences in disease morbidity and mortality between the two groups [22]. A relatively low E-value (2.3) suggests that TB itself might not be the primary driver of mortality in this population. Instead, the history of TB may act as a marker of underlying vulnerability. A syndemic, which refers to the synergistic interaction of two or more diseases or health conditions in a population, involving low socioeconomic status, high TB burden, and prevalent comorbidities, creates a critical vulnerability that drives higher TB mortality rates [2].

The high morbidity and mortality rates among our TB patients indicate that health systems need to pay more attention to TB complications, not just focus on eradicating the *Mycobacterium* [23, 24]. A new approach may include the following aspects. First, systematically integrate monitoring of TB admission and subsequent deaths. Second, develop national clinical guidelines and provide training for healthcare providers on the diagnosis and management of complications. Third, expand access to diagnostic tools and introduce rehabilitation programs at referral centers, with phased implementation within existing TB and service frameworks to ensure feasibility [25].

## Conclusions

In the Thai national database, newly diagnosed and hospitalized drug sensitive pulmonary TB patients experienced higher death rates and were more frequently readmitted with cardiopulmonary diseases compared with hospitalized non-TB patients. This highlights the need for early assessments of these diseases for hospitalized pulmonary TB patients. Preventive and management strategies for those conditions should be integrated into the national TB control programme [26].

## Supplementary Information

Supplementary material 1.

Supplementary material 2.

Supplementary material 3.

Supplementary material 4.

Supplementary material 5.

## Data Availability

All data cannot be shared with the public as they contain sensitive, individual data. Please contact the Thai Health Information Portal (THIP) gate keeper with data access requests: Mr. Nipon Rattankom, Department of Epidemiology, Prince of Songkla University Hatyai, Thailand, nipon.rdh@gmail.com. The Ethics Committee may be contacted at [medpsu.ec@gmail.com] (mailto:medpsu.ec@gmail.com). The sample data are shown in additional file 1.

## Abbreviations

CI: Confidence interval
HR: Hazard ratios
ICD-10: International Classification of Diseases, Tenth Revision
LMICs: Low- and middle-income countries
NHSO: National Health Security Office
SEA: Southeast Asia
TB: Tuberculosis
THIP: Thai Hospital Information Portal
