# Supplementary material for: Readmission and survival of hospitalized pulmonary tuberculosis patients: a nationwide record-based cohort analysis in Thailand (2017–2022)
Source: Infect Dis Poverty. 2026 Jun 15;15:67. doi: 10.1186/s40249-026-01467-0 (PMC13267298; doi:10.1186/s40249-026-01467-0)
Supplement: Supplementary file 1 — Supplementary material 1. [file 40249_2026_1467_MOESM1_ESM.pdf]

TB sequelae

PSU

2024-03-09

```
library(dplyr)
library(lubridate)
library(tidyverse)
```

```
## Warning: package 'ggplot2' was built under R version 4.4.3
```

```
library(data.table)
library(AMR)
```

```
## Warning: package 'AMR' was built under R version 4.4.3
```

```
library(stringr)
library(epiR)
```

```
## Warning: package 'epiR' was built under R version 4.4.3
```

```
library(forcats)
#
library(MatchIt)
library(cobalt)
#For incidence curve
library("survminer")
```

```
## Warning: package 'survminer' was built under R version 4.4.3
```

```
## Warning: package 'ggpubr' was built under R version 4.4.3
```

```
library(ggsurvfit)
```

```
## Warning: package 'ggsurvfit' was built under R version 4.4.3
```

```
library(tidycmprsk)
```

```
## Warning: package 'tidycmprsk' was built under R version 4.4.3
```

```
library(flexsurv)
```

```
## Warning: package 'flexsurv' was built under R version 4.4.3
```

```
library(coxphw)
```

```
## Warning: package 'coxphw' was built under R version 4.4.3
```

```
load("D:\\Post TB\\TB data\\Results\\Journal submission\\Rcodes\\cc.data.RData")
```

```
#Background characteristics of unmatched case and conrol
cc.data$month<-factor(cc.data$month)
cc.data$year<-factor(cc.data$year)
cc.data %>% group_by(group) %>%
summarise(Q1=quantile(age_y,probs = 0.25,na.rm=TRUE),
                    median=median(age_y,na.rm=TRUE), Q3=quantile(age_y, probs = 0.75,na.rm=TRUE))
```

```
## # A tibble: 2 × 4
##   group     Q1 median    Q3
##   <fct>  <dbl>  <int> <dbl>
## 1 Non-TB    51     61    69
## 2 TB        46     58    70
```

```
unmatchcc<-table1::table1(~sex+age_y+year+month+HIV+DM+COPD+CA+HF+IHD|group,data=cc.data)
unmatchcc
```

	Non-TB (N=459755)	TB (N=141937)	Overall (N=601692)
**sex**			
Female	133631 (29.1%)	39488 (27.8%)	173119 (28.8%)
Male	326124 (70.9%)	102449 (72.2%)	428573 (71.2%)
**age_y**			
Mean (SD)	59.4 (13.6)	57.1 (16.9)	58.9 (14.5)
Median [Min, Max]	61.0 [15.0, 106]	58.0 [15.0, 112]	60.0 [15.0, 112]
**year**			
2015	4 (0.0%)	0 (0%)	4 (0.0%)
2016	22789 (5.0%)	0 (0%)	22789 (3.8%)
2017	103336 (22.5%)	26800 (18.9%)	130136 (21.6%)
2018	88536 (19.3%)	26504 (18.7%)	115040 (19.1%)
2019	81292 (17.7%)	26106 (18.4%)	107398 (17.8%)
2020	67768 (14.7%)	25068 (17.7%)	92836 (15.4%)
2021	58437 (12.7%)	22166 (15.6%)	80603 (13.4%)
2022	37593 (8.2%)	15293 (10.8%)	52886 (8.8%)
**month**			
1	42637 (9.3%)	13023 (9.2%)	55660 (9.3%)
2	38777 (8.4%)	12373 (8.7%)	51150 (8.5%)
3	42202 (9.2%)	13889 (9.8%)	56091 (9.3%)
4	35712 (7.8%)	12365 (8.7%)	48077 (8.0%)
5	38235 (8.3%)	13031 (9.2%)	51266 (8.5%)
6	38143 (8.3%)	12343 (8.7%)	50486 (8.4%)
7	36466 (7.9%)	11594 (8.2%)	48060 (8.0%)
8	37236 (8.1%)	11908 (8.4%)	49144 (8.2%)
9	35508 (7.7%)	11352 (8.0%)	46860 (7.8%)
10	41132 (8.9%)	10367 (7.3%)	51499 (8.6%)
11	37966 (8.3%)	10100 (7.1%)	48066 (8.0%)
12	35741 (7.8%)	9592 (6.8%)	45333 (7.5%)
**HIV**			
HIV	10081 (2.2%)	4144 (2.9%)	14225 (2.4%)
NO	449674 (97.8%)	137793 (97.1%)	587467 (97.6%)
**DM**			
DM	170502 (37.1%)	39248 (27.7%)	209750 (34.9%)

	Non-TB (N=459755)	TB (N=141937)	Overall (N=601692)
NO	289253 (62.9%)	102689 (72.3%)	391942 (65.1%)
**COPD**			
COPD	40511 (8.8%)	9972 (7.0%)	50483 (8.4%)
NO	419244 (91.2%)	131965 (93.0%)	551209 (91.6%)
**CA**			
CA	86868 (18.9%)	8414 (5.9%)	95282 (15.8%)
NO	372887 (81.1%)	133523 (94.1%)	506410 (84.2%)
**HF**			
HF	67517 (14.7%)	7167 (5.0%)	74684 (12.4%)
NO	392238 (85.3%)	134770 (95.0%)	527008 (87.6%)
**IHD**			
IHD	271832 (59.1%)	56874 (40.1%)	328706 (54.6%)
NO	187923 (40.9%)	85063 (59.9%)	272986 (45.4%)

#Propensity score matching

```
#cc.data$superdistrict<-factor(cc.data$superdistrict)
#cc.data$group<-factor(cc.data$group)
#cc.data$day<-factor(cc.data$day)
#cc.data$month<-factor(cc.data$month)
#cc.data$year<-factor(cc.data$year)
#cc.data$DM<-factor(cc.data$DM)
#cc.data$HIV<-factor(cc.data$HIV)
#cc.data$CA<-factor(cc.data$CA)
#cc.data$COPD<-factor(cc.data$COPD)
#cc.data$HF<-factor(cc.data$HF)
#cc.data$IHD<-factor(cc.data$IHD)
#m.out12 <- matchit(group~sex+age_y+DM+HIV+CA+COPD+HF+IHD,
 #                data=cc.data,
  #             method = "nearest",
   #             caliper=0.2,
    #            ratio=1,
#exact = ~ superdistrict+day+month+year)
```

```
#save(m.out12,file="D:\\Post TB\\TB data\\Results\\Journal submission\\Rcodes\\m.out12.Rdata")
load("D:\\Post TB\\TB data\\Results\\Journal submission\\Rcodes\\m.out12.Rdata")
```

```
#Standardized mean difference foe un-matched data
head(summary(m.out12)$sum.all,20)
```

```
##                 Means Treated Means Control Std. Mean Diff. Var. Ratio
## distance         2.720502e-01  2.247349e-01      0.66951647  0.5534813
## sexFemale        2.782079e-01  2.906570e-01     -0.02778083         NA
## sexMale          7.217921e-01  7.093430e-01      0.02778083         NA
## age_y            5.713505e+01  5.941962e+01     -0.13511910  1.5494177
## DMDM             2.765170e-01  3.708540e-01     -0.21091474         NA
## DMNO             7.234830e-01  6.291460e-01      0.21091474         NA
## HIVHIV           2.919605e-02  2.192690e-02      0.04317737         NA
## HIVNO            9.708039e-01  9.780731e-01     -0.04317737         NA
## CACA             5.927982e-02  1.889441e-01     -0.54908138         NA
## CANO             9.407202e-01  8.110559e-01      0.54908138         NA
## COPDCOPD         7.025652e-02  8.811432e-02     -0.06987198         NA
## COPDNO           9.297435e-01  9.118857e-01      0.06987198         NA
## HFHF             5.049423e-02  1.468543e-01     -0.44007572         NA
## HFNO             9.495058e-01  8.531457e-01      0.44007572         NA
## IHDIHD           4.006989e-01  5.912540e-01     -0.38885622         NA
## IHDNO            5.993011e-01  4.087460e-01      0.38885622         NA
## superdistrict1  8.243094e-04  2.501332e-04      0.02000687         NA
## superdistrict2  5.918119e-04  1.305043e-04      0.01896825         NA
## superdistrict4  1.317486e-03  4.415395e-04      0.02414854         NA
## superdistrict5  6.693110e-04  1.457298e-04      0.02024489         NA
##                    eCDF Mean     eCDF Max Std. Pair Dist.
## distance       0.1243314232 0.2299094971              NA
## sexFemale      0.0124490430 0.0124490430              NA
## sexMale        0.0124490430 0.0124490430              NA
## age_y          0.0382661750 0.1075887958              NA
## DMDM           0.0943369955 0.0943369955              NA
## DMNO           0.0943369955 0.0943369955              NA
## HIVHIV         0.0072691559 0.0072691559              NA
## HIVNO          0.0072691559 0.0072691559              NA
## CACA           0.1296642902 0.1296642902              NA
## CANO           0.1296642902 0.1296642902              NA
## COPDCOPD       0.0178577995 0.0178577995              NA
## COPDNO         0.0178577995 0.0178577995              NA
## HFHF           0.0963600695 0.0963600695              NA
## HFNO           0.0963600695 0.0963600695              NA
## IHDIHD         0.1905551359 0.1905551359              NA
## IHDNO          0.1905551359 0.1905551359              NA
## superdistrict1 0.0005741762 0.0005741762              NA
## superdistrict2 0.0004613076 0.0004613076              NA
## superdistrict4 0.0008759464 0.0008759464              NA
## superdistrict5 0.0005235812 0.0005235812              NA
```

```
#Standardized mean difference foe un-matched data
tail(summary(m.out12)$sum.all,20)
```

```
##          Means Treated Means Control Std. Mean Diff. Var. Ratio    eCDF Mean
## month1      0.09175197  9.273852e-02    -0.003417503         NA 9.865493e-04
## month2      0.08717248  8.434275e-02     0.010031387         NA 2.829730e-03
## month3      0.09785327  9.179237e-02     0.020399110         NA 6.060905e-03
## month4      0.08711611  7.767615e-02     0.033474430         NA 9.439961e-03
## month5      0.09180834  8.316386e-02     0.029937049         NA 8.644478e-03
## month6      0.08696112  8.296375e-02     0.014186214         NA 3.997364e-03
## month7      0.08168413  7.931616e-02     0.008645922         NA 2.367970e-03
## month8      0.08389638  8.099096e-02     0.010480068         NA 2.905414e-03
## month9      0.07997915  7.723244e-02     0.010125700         NA 2.746707e-03
## month10     0.07303945  8.946504e-02    -0.063126502         NA 1.642559e-02
## month11     0.07115833  8.257876e-02    -0.044422080         NA 1.142043e-02
## month12     0.06757928  7.773923e-02    -0.040474263         NA 1.015995e-02
## year2015    0.00000000  8.700286e-06    -0.003374369         NA 8.700286e-06
## year2016    0.00000000  4.956770e-02    -0.261253850         NA 4.956770e-02
## year2017    0.18881616  2.247632e-01    -0.091850963         NA 3.594702e-02
## year2018    0.18673073  1.925721e-01    -0.014989658         NA 5.841398e-03
## year2019    0.18392667  1.768159e-01     0.018353927         NA 7.110759e-03
## year2020    0.17661357  1.474002e-01     0.076606754         NA 2.921332e-02
## year2021    0.15616788  1.271047e-01     0.080060796         NA 2.906322e-02
## year2022    0.10774499  8.176746e-02     0.083782766         NA 2.597752e-02
##              eCDF Max Std. Pair Dist.
## month1   9.865493e-04              NA
## month2   2.829730e-03              NA
## month3   6.060905e-03              NA
## month4   9.439961e-03              NA
## month5   8.644478e-03              NA
## month6   3.997364e-03              NA
## month7   2.367970e-03              NA
## month8   2.905414e-03              NA
## month9   2.746707e-03              NA
## month10  1.642559e-02              NA
## month11  1.142043e-02              NA
## month12  1.015995e-02              NA
## year2015 8.700286e-06              NA
## year2016 4.956770e-02              NA
## year2017 3.594702e-02              NA
## year2018 5.841398e-03              NA
## year2019 7.110759e-03              NA
## year2020 2.921332e-02              NA
## year2021 2.906322e-02              NA
## year2022 2.597752e-02              NA
```

```
#cc.match2 <- match.data(m.out12)
#save(cc.match2,file="D:\\Post TB\\TB data\\Results\\Journal submission\\Rcodes\\cc.match2.Rdata")
#Skip including death into cc.match1
```

```
load("D:\\Post TB\\TB data\\Results\\Journal submission\\Rcodes\\cc.match2.RData")
matchcc<-table1::table1(~sex+age_y+year+month+HIV+DM+COPD+CA+HF+IHD|group,data=cc.match2)
matchcc
```

	Non-TB (N=59027)	TB (N=59027)	Overall (N=118054)
**sex**			
Female	16201 (27.4%)	16184 (27.4%)	32385 (27.4%)
Male	42826 (72.6%)	42843 (72.6%)	85669 (72.6%)
**age_y**			
Mean (SD)	57.1 (15.2)	57.0 (15.6)	57.0 (15.4)
Median [Min, Max]	59.0 [15.0, 104]	58.0 [15.0, 103]	58.0 [15.0, 104]
**year**			
2015	0 (0%)	0 (0%)	0 (0%)

	Non-TB (N=59027)	TB (N=59027)	Overall (N=118054)
2016	0 (0%)	0 (0%)	0 (0%)
2017	11802 (20.0%)	11802 (20.0%)	23604 (20.0%)
2018	11387 (19.3%)	11387 (19.3%)	22774 (19.3%)
2019	10942 (18.5%)	10942 (18.5%)	21884 (18.5%)
2020	9778 (16.6%)	9778 (16.6%)	19556 (16.6%)
2021	8891 (15.1%)	8891 (15.1%)	17782 (15.1%)
2022	6227 (10.5%)	6227 (10.5%)	12454 (10.5%)
**month**			
1	5469 (9.3%)	5469 (9.3%)	10938 (9.3%)
2	5117 (8.7%)	5117 (8.7%)	10234 (8.7%)
3	5888 (10.0%)	5888 (10.0%)	11776 (10.0%)
4	4970 (8.4%)	4970 (8.4%)	9940 (8.4%)
5	5319 (9.0%)	5319 (9.0%)	10638 (9.0%)
6	5227 (8.9%)	5227 (8.9%)	10454 (8.9%)
7	4853 (8.2%)	4853 (8.2%)	9706 (8.2%)
8	5047 (8.6%)	5047 (8.6%)	10094 (8.6%)
9	4703 (8.0%)	4703 (8.0%)	9406 (8.0%)
10	4425 (7.5%)	4425 (7.5%)	8850 (7.5%)
11	4208 (7.1%)	4208 (7.1%)	8416 (7.1%)
12	3801 (6.4%)	3801 (6.4%)	7602 (6.4%)
**HIV**			
HIV	418 (0.7%)	578 (1.0%)	996 (0.8%)
NO	58609 (99.3%)	58449 (99.0%)	117058 (99.2%)
**DM**			
DM	12407 (21.0%)	12701 (21.5%)	25108 (21.3%)
NO	46620 (79.0%)	46326 (78.5%)	92946 (78.7%)
**COPD**			
COPD	1817 (3.1%)	2169 (3.7%)	3986 (3.4%)
NO	57210 (96.9%)	56858 (96.3%)	114068 (96.6%)
**CA**			
CA	2809 (4.8%)	2595 (4.4%)	5404 (4.6%)
NO	56218 (95.2%)	56432 (95.6%)	112650 (95.4%)
**HF**			
HF	1688 (2.9%)	1902 (3.2%)	3590 (3.0%)
NO	57339 (97.1%)	57125 (96.8%)	114464 (97.0%)
**IHD**			
IHD	16247 (27.5%)	16446 (27.9%)	32693 (27.7%)
NO	42780 (72.5%)	42581 (72.1%)	85361 (72.3%)

```
#Standardized mean difference for matched data
head(summary(m.out12)$sum.matched,20)
```

```
##                Means Treated Means Control Std. Mean Diff. Var. Ratio
## distance        2.823684e-01  2.822238e-01    0.0020458894   1.001050
## sexFemale       2.741796e-01  2.744676e-01   -0.0006426987         NA
## sexMale         7.258204e-01  7.255324e-01    0.0006426987         NA
## age_y           5.697664e+01  5.706938e+01   -0.0054848741   1.056229
## DMDM            2.151727e-01  2.101919e-01    0.0111358023         NA
## DMNO            7.848273e-01  7.898081e-01   -0.0111358023         NA
## HIVHIV          9.792129e-03  7.081505e-03    0.0161005771         NA
## HIVNO           9.902079e-01  9.929185e-01   -0.0161005771         NA
## CACA            4.396293e-02  4.758839e-02   -0.0153525100         NA
## CANO            9.560371e-01  9.524116e-01    0.0153525100         NA
## COPDCOPD        3.674590e-02  3.078252e-02    0.0233328107         NA
## COPDNO          9.632541e-01  9.692175e-01   -0.0233328107         NA
## HFHF            3.222254e-02  2.859708e-02    0.0165574468         NA
## HFNO            9.677775e-01  9.714029e-01   -0.0165574468         NA
## IHDIHD          2.786183e-01  2.752469e-01    0.0068797198         NA
## IHDNO           7.213817e-01  7.247531e-01   -0.0068797198         NA
## superdistrict1  2.202382e-04  2.202382e-04    0.0000000000         NA
## superdistrict2  1.355312e-04  1.355312e-04    0.0000000000         NA
## superdistrict4  4.574178e-04  4.574178e-04    0.0000000000         NA
## superdistrict5  8.470700e-05  8.470700e-05    0.0000000000         NA
##                   eCDF Mean     eCDF Max Std. Pair Dist.
## distance       0.0013160788 0.0057261931      0.04291906
## sexFemale      0.0002880038 0.0002880038      0.19209129
## sexMale        0.0002880038 0.0002880038      0.19209129
## age_y          0.0049454769 0.0134514714      0.19429320
## DMDM           0.0049807715 0.0049807715      0.12006971
## DMNO           0.0049807715 0.0049807715      0.12006971
## HIVHIV         0.0027106240 0.0027106240      0.08231420
## HIVNO          0.0027106240 0.0027106240      0.08231420
## CACA           0.0036254595 0.0036254595      0.13602037
## CANO           0.0036254595 0.0036254595      0.13602037
## COPDCOPD       0.0059633727 0.0059633727      0.19037452
## COPDNO         0.0059633727 0.0059633727      0.19037452
## HFHF           0.0036254595 0.0036254595      0.14669588
## HFNO           0.0036254595 0.0036254595      0.14669588
## IHDIHD         0.0033713385 0.0033713385      0.04380204
## IHDNO          0.0033713385 0.0033713385      0.04380204
## superdistrict1 0.0000000000 0.0000000000      0.00000000
## superdistrict2 0.0000000000 0.0000000000      0.00000000
## superdistrict4 0.0000000000 0.0000000000      0.00000000
## superdistrict5 0.0000000000 0.0000000000      0.00000000
```

```
#Standardized mean difference after matching
tail(summary(m.out12)$sum.matched,20)
```

```
##          Means Treated Means Control Std. Mean Diff. Var. Ratio eCDF Mean
## month1      0.09265251    0.09265251               0         NA         0
## month2      0.08668914    0.08668914               0         NA         0
## month3      0.09975096    0.09975096               0         NA         0
## month4      0.08419876    0.08419876               0         NA         0
## month5      0.09011130    0.09011130               0         NA         0
## month6      0.08855270    0.08855270               0         NA         0
## month7      0.08221661    0.08221661               0         NA         0
## month8      0.08550324    0.08550324               0         NA         0
## month9      0.07967540    0.07967540               0         NA         0
## month10     0.07496569    0.07496569               0         NA         0
## month11     0.07128941    0.07128941               0         NA         0
## month12     0.06439426    0.06439426               0         NA         0
## year2015    0.00000000    0.00000000               0         NA         0
## year2016    0.00000000    0.00000000               0         NA         0
## year2017    0.19994240    0.19994240               0         NA         0
## year2018    0.19291172    0.19291172               0         NA         0
## year2019    0.18537280    0.18537280               0         NA         0
## year2020    0.16565301    0.16565301               0         NA         0
## year2021    0.15062598    0.15062598               0         NA         0
## year2022    0.10549410    0.10549410               0         NA         0
##          eCDF Max Std. Pair Dist.
## month1          0               0
## month2          0               0
## month3          0               0
## month4          0               0
## month5          0               0
## month6          0               0
## month7          0               0
## month8          0               0
## month9          0               0
## month10         0               0
## month11         0               0
## month12         0               0
## year2015        0               0
## year2016        0               0
## year2017        0               0
## year2018        0               0
## year2019        0               0
## year2020        0               0
## year2021        0               0
## year2022        0               0
```

```
#Checking balance of covariates
bal.tab(m.out12,m.threshold=0.1)
```

```
## Balance Measures
##                       Type Diff.Adj    M.Threshold
## distance          Distance   0.0020 Balanced, <0.1
## sex_Male            Binary   0.0003 Balanced, <0.1
## age_y              Contin.  -0.0055 Balanced, <0.1
## DM_NO               Binary  -0.0050 Balanced, <0.1
## HIV_NO              Binary  -0.0027 Balanced, <0.1
## CA_NO               Binary   0.0036 Balanced, <0.1
## COPD_NO             Binary  -0.0060 Balanced, <0.1
## HF_NO               Binary  -0.0036 Balanced, <0.1
## IHD_NO              Binary  -0.0034 Balanced, <0.1
## superdistrict_1     Binary   0.0000 Balanced, <0.1
## superdistrict_2     Binary   0.0000 Balanced, <0.1
## superdistrict_4     Binary   0.0000 Balanced, <0.1
## superdistrict_5     Binary   0.0000 Balanced, <0.1
## superdistrict_6     Binary   0.0000 Balanced, <0.1
## superdistrict_7     Binary   0.0000 Balanced, <0.1
## superdistrict_8     Binary   0.0000 Balanced, <0.1
## superdistrict_9     Binary   0.0000 Balanced, <0.1
## superdistrict_10    Binary   0.0000 Balanced, <0.1
## superdistrict_11    Binary   0.0000 Balanced, <0.1
## superdistrict_12    Binary   0.0000 Balanced, <0.1
## superdistrict_13    Binary   0.0000 Balanced, <0.1
## superdistrict_14    Binary   0.0000 Balanced, <0.1
## superdistrict_16    Binary   0.0000 Balanced, <0.1
## superdistrict_17    Binary   0.0000 Balanced, <0.1
## superdistrict_18    Binary   0.0000 Balanced, <0.1
## superdistrict_21    Binary   0.0000 Balanced, <0.1
## superdistrict_22    Binary   0.0000 Balanced, <0.1
## superdistrict_23    Binary   0.0000 Balanced, <0.1
## superdistrict_24    Binary   0.0000 Balanced, <0.1
## superdistrict_25    Binary   0.0000 Balanced, <0.1
## superdistrict_27    Binary   0.0000 Balanced, <0.1
## superdistrict_28    Binary   0.0000 Balanced, <0.1
## superdistrict_29    Binary   0.0000 Balanced, <0.1
## superdistrict_30    Binary   0.0000 Balanced, <0.1
## superdistrict_31    Binary   0.0000 Balanced, <0.1
## superdistrict_32    Binary   0.0000 Balanced, <0.1
## superdistrict_33    Binary   0.0000 Balanced, <0.1
## superdistrict_34    Binary   0.0000 Balanced, <0.1
## superdistrict_35    Binary   0.0000 Balanced, <0.1
## superdistrict_36    Binary   0.0000 Balanced, <0.1
## superdistrict_37    Binary   0.0000 Balanced, <0.1
## superdistrict_38    Binary   0.0000 Balanced, <0.1
## superdistrict_39    Binary   0.0000 Balanced, <0.1
## superdistrict_40    Binary   0.0000 Balanced, <0.1
## superdistrict_41    Binary   0.0000 Balanced, <0.1
## superdistrict_42    Binary   0.0000 Balanced, <0.1
## superdistrict_43    Binary   0.0000 Balanced, <0.1
## superdistrict_44    Binary   0.0000 Balanced, <0.1
## superdistrict_45    Binary   0.0000 Balanced, <0.1
## superdistrict_46    Binary   0.0000 Balanced, <0.1
## superdistrict_47    Binary   0.0000 Balanced, <0.1
## superdistrict_48    Binary   0.0000 Balanced, <0.1
## superdistrict_49    Binary   0.0000 Balanced, <0.1
## superdistrict_50    Binary   0.0000 Balanced, <0.1
## superdistrict_51    Binary   0.0000 Balanced, <0.1
## superdistrict_52    Binary   0.0000 Balanced, <0.1
## superdistrict_53    Binary   0.0000 Balanced, <0.1
## superdistrict_54    Binary   0.0000 Balanced, <0.1
## superdistrict_55    Binary   0.0000 Balanced, <0.1
## superdistrict_56    Binary   0.0000 Balanced, <0.1
## superdistrict_57    Binary   0.0000 Balanced, <0.1
## superdistrict_58    Binary   0.0000 Balanced, <0.1
## superdistrict_59    Binary   0.0000 Balanced, <0.1
## superdistrict_60    Binary   0.0000 Balanced, <0.1
## superdistrict_61    Binary   0.0000 Balanced, <0.1
```

```
## superdistrict_62    Binary   0.0000 Balanced, <0.1
## superdistrict_63    Binary   0.0000 Balanced, <0.1
## superdistrict_64    Binary   0.0000 Balanced, <0.1
## superdistrict_65    Binary   0.0000 Balanced, <0.1
## superdistrict_66    Binary   0.0000 Balanced, <0.1
## superdistrict_67    Binary   0.0000 Balanced, <0.1
## superdistrict_68    Binary   0.0000 Balanced, <0.1
## superdistrict_69    Binary   0.0000 Balanced, <0.1
## superdistrict_70    Binary   0.0000 Balanced, <0.1
## superdistrict_71    Binary   0.0000 Balanced, <0.1
## superdistrict_72    Binary   0.0000 Balanced, <0.1
## superdistrict_73    Binary   0.0000 Balanced, <0.1
## superdistrict_74    Binary   0.0000 Balanced, <0.1
## superdistrict_75    Binary   0.0000 Balanced, <0.1
## superdistrict_76    Binary   0.0000 Balanced, <0.1
## superdistrict_77    Binary   0.0000 Balanced, <0.1
## superdistrict_78    Binary   0.0000 Balanced, <0.1
## superdistrict_79    Binary   0.0000 Balanced, <0.1
## superdistrict_80    Binary   0.0000 Balanced, <0.1
## superdistrict_81    Binary   0.0000 Balanced, <0.1
## superdistrict_82    Binary   0.0000 Balanced, <0.1
## superdistrict_83    Binary   0.0000 Balanced, <0.1
## superdistrict_84    Binary   0.0000 Balanced, <0.1
## superdistrict_85    Binary   0.0000 Balanced, <0.1
## superdistrict_86    Binary   0.0000 Balanced, <0.1
## superdistrict_87    Binary   0.0000 Balanced, <0.1
## superdistrict_88    Binary   0.0000 Balanced, <0.1
## superdistrict_89    Binary   0.0000 Balanced, <0.1
## superdistrict_90    Binary   0.0000 Balanced, <0.1
## superdistrict_91    Binary   0.0000 Balanced, <0.1
## superdistrict_92    Binary   0.0000 Balanced, <0.1
## superdistrict_93    Binary   0.0000 Balanced, <0.1
## superdistrict_94    Binary   0.0000 Balanced, <0.1
## superdistrict_95    Binary   0.0000 Balanced, <0.1
## superdistrict_96    Binary   0.0000 Balanced, <0.1
## superdistrict_97    Binary   0.0000 Balanced, <0.1
## superdistrict_98    Binary   0.0000 Balanced, <0.1
## superdistrict_99    Binary   0.0000 Balanced, <0.1
## superdistrict_100   Binary   0.0000 Balanced, <0.1
## superdistrict_101   Binary   0.0000 Balanced, <0.1
## superdistrict_102   Binary   0.0000 Balanced, <0.1
## superdistrict_103   Binary   0.0000 Balanced, <0.1
## superdistrict_104   Binary   0.0000 Balanced, <0.1
## superdistrict_105   Binary   0.0000 Balanced, <0.1
## superdistrict_106   Binary   0.0000 Balanced, <0.1
## superdistrict_107   Binary   0.0000 Balanced, <0.1
## superdistrict_108   Binary   0.0000 Balanced, <0.1
## superdistrict_109   Binary   0.0000 Balanced, <0.1
## superdistrict_110   Binary   0.0000 Balanced, <0.1
## superdistrict_111   Binary   0.0000 Balanced, <0.1
## superdistrict_112   Binary   0.0000 Balanced, <0.1
## superdistrict_113   Binary   0.0000 Balanced, <0.1
## superdistrict_114   Binary   0.0000 Balanced, <0.1
## superdistrict_115   Binary   0.0000 Balanced, <0.1
## superdistrict_116   Binary   0.0000 Balanced, <0.1
## superdistrict_117   Binary   0.0000 Balanced, <0.1
## superdistrict_118   Binary   0.0000 Balanced, <0.1
## superdistrict_119   Binary   0.0000 Balanced, <0.1
## superdistrict_120   Binary   0.0000 Balanced, <0.1
## superdistrict_121   Binary   0.0000 Balanced, <0.1
## superdistrict_122   Binary   0.0000 Balanced, <0.1
## superdistrict_123   Binary   0.0000 Balanced, <0.1
## superdistrict_124   Binary   0.0000 Balanced, <0.1
## superdistrict_125   Binary   0.0000 Balanced, <0.1
## superdistrict_126   Binary   0.0000 Balanced, <0.1
## superdistrict_127   Binary   0.0000 Balanced, <0.1
## superdistrict_128   Binary   0.0000 Balanced, <0.1
```

```
## superdistrict_129   Binary   0.0000 Balanced, <0.1
## superdistrict_130   Binary   0.0000 Balanced, <0.1
## superdistrict_131   Binary   0.0000 Balanced, <0.1
## superdistrict_132   Binary   0.0000 Balanced, <0.1
## superdistrict_133   Binary   0.0000 Balanced, <0.1
## superdistrict_134   Binary   0.0000 Balanced, <0.1
## superdistrict_135   Binary   0.0000 Balanced, <0.1
## superdistrict_136   Binary   0.0000 Balanced, <0.1
## superdistrict_137   Binary   0.0000 Balanced, <0.1
## superdistrict_138   Binary   0.0000 Balanced, <0.1
## superdistrict_139   Binary   0.0000 Balanced, <0.1
## superdistrict_140   Binary   0.0000 Balanced, <0.1
## superdistrict_141   Binary   0.0000 Balanced, <0.1
## superdistrict_142   Binary   0.0000 Balanced, <0.1
## superdistrict_143   Binary   0.0000 Balanced, <0.1
## superdistrict_144   Binary   0.0000 Balanced, <0.1
## superdistrict_145   Binary   0.0000 Balanced, <0.1
## superdistrict_146   Binary   0.0000 Balanced, <0.1
## superdistrict_147   Binary   0.0000 Balanced, <0.1
## superdistrict_148   Binary   0.0000 Balanced, <0.1
## superdistrict_149   Binary   0.0000 Balanced, <0.1
## superdistrict_150   Binary   0.0000 Balanced, <0.1
## superdistrict_151   Binary   0.0000 Balanced, <0.1
## superdistrict_152   Binary   0.0000 Balanced, <0.1
## superdistrict_153   Binary   0.0000 Balanced, <0.1
## superdistrict_154   Binary   0.0000 Balanced, <0.1
## superdistrict_155   Binary   0.0000 Balanced, <0.1
## superdistrict_156   Binary   0.0000 Balanced, <0.1
## superdistrict_157   Binary   0.0000 Balanced, <0.1
## superdistrict_158   Binary   0.0000 Balanced, <0.1
## superdistrict_159   Binary   0.0000 Balanced, <0.1
## superdistrict_160   Binary   0.0000 Balanced, <0.1
## superdistrict_161   Binary   0.0000 Balanced, <0.1
## superdistrict_162   Binary   0.0000 Balanced, <0.1
## superdistrict_163   Binary   0.0000 Balanced, <0.1
## superdistrict_164   Binary   0.0000 Balanced, <0.1
## superdistrict_165   Binary   0.0000 Balanced, <0.1
## superdistrict_166   Binary   0.0000 Balanced, <0.1
## superdistrict_167   Binary   0.0000 Balanced, <0.1
## superdistrict_168   Binary   0.0000 Balanced, <0.1
## superdistrict_169   Binary   0.0000 Balanced, <0.1
## superdistrict_170   Binary   0.0000 Balanced, <0.1
## superdistrict_171   Binary   0.0000 Balanced, <0.1
## superdistrict_172   Binary   0.0000 Balanced, <0.1
## superdistrict_173   Binary   0.0000 Balanced, <0.1
## superdistrict_174   Binary   0.0000 Balanced, <0.1
## superdistrict_175   Binary   0.0000 Balanced, <0.1
## superdistrict_176   Binary   0.0000 Balanced, <0.1
## superdistrict_177   Binary   0.0000 Balanced, <0.1
## superdistrict_178   Binary   0.0000 Balanced, <0.1
## superdistrict_179   Binary   0.0000 Balanced, <0.1
## superdistrict_180   Binary   0.0000 Balanced, <0.1
## superdistrict_181   Binary   0.0000 Balanced, <0.1
## superdistrict_182   Binary   0.0000 Balanced, <0.1
## superdistrict_183   Binary   0.0000 Balanced, <0.1
## superdistrict_184   Binary   0.0000 Balanced, <0.1
## superdistrict_185   Binary   0.0000 Balanced, <0.1
## superdistrict_186   Binary   0.0000 Balanced, <0.1
## superdistrict_187   Binary   0.0000 Balanced, <0.1
## superdistrict_188   Binary   0.0000 Balanced, <0.1
## superdistrict_189   Binary   0.0000 Balanced, <0.1
## superdistrict_190   Binary   0.0000 Balanced, <0.1
## superdistrict_191   Binary   0.0000 Balanced, <0.1
## superdistrict_192   Binary   0.0000 Balanced, <0.1
## superdistrict_193   Binary   0.0000 Balanced, <0.1
## superdistrict_194   Binary   0.0000 Balanced, <0.1
## superdistrict_195   Binary   0.0000 Balanced, <0.1
```

```
## superdistrict_196   Binary   0.0000 Balanced, <0.1
## superdistrict_197   Binary   0.0000 Balanced, <0.1
## superdistrict_198   Binary   0.0000 Balanced, <0.1
## superdistrict_199   Binary   0.0000 Balanced, <0.1
## superdistrict_200   Binary   0.0000 Balanced, <0.1
## superdistrict_201   Binary   0.0000 Balanced, <0.1
## superdistrict_202   Binary   0.0000 Balanced, <0.1
## superdistrict_203   Binary   0.0000 Balanced, <0.1
## superdistrict_204   Binary   0.0000 Balanced, <0.1
## superdistrict_205   Binary   0.0000 Balanced, <0.1
## superdistrict_206   Binary   0.0000 Balanced, <0.1
## superdistrict_207   Binary   0.0000 Balanced, <0.1
## superdistrict_208   Binary   0.0000 Balanced, <0.1
## superdistrict_209   Binary   0.0000 Balanced, <0.1
## superdistrict_210   Binary   0.0000 Balanced, <0.1
## superdistrict_211   Binary   0.0000 Balanced, <0.1
## superdistrict_212   Binary   0.0000 Balanced, <0.1
## superdistrict_213   Binary   0.0000 Balanced, <0.1
## superdistrict_214   Binary   0.0000 Balanced, <0.1
## superdistrict_215   Binary   0.0000 Balanced, <0.1
## superdistrict_216   Binary   0.0000 Balanced, <0.1
## superdistrict_217   Binary   0.0000 Balanced, <0.1
## superdistrict_218   Binary   0.0000 Balanced, <0.1
## superdistrict_219   Binary   0.0000 Balanced, <0.1
## superdistrict_220   Binary   0.0000 Balanced, <0.1
## superdistrict_221   Binary   0.0000 Balanced, <0.1
## superdistrict_222   Binary   0.0000 Balanced, <0.1
## superdistrict_223   Binary   0.0000 Balanced, <0.1
## superdistrict_224   Binary   0.0000 Balanced, <0.1
## superdistrict_225   Binary   0.0000 Balanced, <0.1
## superdistrict_226   Binary   0.0000 Balanced, <0.1
## superdistrict_227   Binary   0.0000 Balanced, <0.1
## superdistrict_228   Binary   0.0000 Balanced, <0.1
## superdistrict_229   Binary   0.0000 Balanced, <0.1
## superdistrict_230   Binary   0.0000 Balanced, <0.1
## superdistrict_231   Binary   0.0000 Balanced, <0.1
## superdistrict_232   Binary   0.0000 Balanced, <0.1
## superdistrict_233   Binary   0.0000 Balanced, <0.1
## superdistrict_234   Binary   0.0000 Balanced, <0.1
## superdistrict_235   Binary   0.0000 Balanced, <0.1
## superdistrict_236   Binary   0.0000 Balanced, <0.1
## superdistrict_237   Binary   0.0000 Balanced, <0.1
## superdistrict_238   Binary   0.0000 Balanced, <0.1
## superdistrict_239   Binary   0.0000 Balanced, <0.1
## superdistrict_240   Binary   0.0000 Balanced, <0.1
## superdistrict_241   Binary   0.0000 Balanced, <0.1
## superdistrict_242   Binary   0.0000 Balanced, <0.1
## superdistrict_243   Binary   0.0000 Balanced, <0.1
## superdistrict_244   Binary   0.0000 Balanced, <0.1
## superdistrict_245   Binary   0.0000 Balanced, <0.1
## superdistrict_246   Binary   0.0000 Balanced, <0.1
## superdistrict_247   Binary   0.0000 Balanced, <0.1
## superdistrict_248   Binary   0.0000 Balanced, <0.1
## superdistrict_249   Binary   0.0000 Balanced, <0.1
## superdistrict_250   Binary   0.0000 Balanced, <0.1
## superdistrict_251   Binary   0.0000 Balanced, <0.1
## superdistrict_252   Binary   0.0000 Balanced, <0.1
## superdistrict_253   Binary   0.0000 Balanced, <0.1
## superdistrict_254   Binary   0.0000 Balanced, <0.1
## superdistrict_255   Binary   0.0000 Balanced, <0.1
## superdistrict_256   Binary   0.0000 Balanced, <0.1
## superdistrict_257   Binary   0.0000 Balanced, <0.1
## superdistrict_258   Binary   0.0000 Balanced, <0.1
## superdistrict_259   Binary   0.0000 Balanced, <0.1
## superdistrict_260   Binary   0.0000 Balanced, <0.1
## superdistrict_261   Binary   0.0000 Balanced, <0.1
## superdistrict_262   Binary   0.0000 Balanced, <0.1
```

```
## superdistrict_263   Binary   0.0000 Balanced, <0.1
## superdistrict_264   Binary   0.0000 Balanced, <0.1
## superdistrict_265   Binary   0.0000 Balanced, <0.1
## superdistrict_266   Binary   0.0000 Balanced, <0.1
## superdistrict_267   Binary   0.0000 Balanced, <0.1
## superdistrict_268   Binary   0.0000 Balanced, <0.1
## superdistrict_269   Binary   0.0000 Balanced, <0.1
## superdistrict_270   Binary   0.0000 Balanced, <0.1
## superdistrict_271   Binary   0.0000 Balanced, <0.1
## superdistrict_272   Binary   0.0000 Balanced, <0.1
## superdistrict_273   Binary   0.0000 Balanced, <0.1
## superdistrict_274   Binary   0.0000 Balanced, <0.1
## superdistrict_275   Binary   0.0000 Balanced, <0.1
## superdistrict_276   Binary   0.0000 Balanced, <0.1
## superdistrict_277   Binary   0.0000 Balanced, <0.1
## superdistrict_278   Binary   0.0000 Balanced, <0.1
## superdistrict_279   Binary   0.0000 Balanced, <0.1
## superdistrict_280   Binary   0.0000 Balanced, <0.1
## superdistrict_281   Binary   0.0000 Balanced, <0.1
## superdistrict_282   Binary   0.0000 Balanced, <0.1
## superdistrict_283   Binary   0.0000 Balanced, <0.1
## superdistrict_284   Binary   0.0000 Balanced, <0.1
## superdistrict_285   Binary   0.0000 Balanced, <0.1
## superdistrict_286   Binary   0.0000 Balanced, <0.1
## superdistrict_287   Binary   0.0000 Balanced, <0.1
## superdistrict_288   Binary   0.0000 Balanced, <0.1
## superdistrict_289   Binary   0.0000 Balanced, <0.1
## superdistrict_290   Binary   0.0000 Balanced, <0.1
## superdistrict_291   Binary   0.0000 Balanced, <0.1
## superdistrict_292   Binary   0.0000 Balanced, <0.1
## superdistrict_293   Binary   0.0000 Balanced, <0.1
## superdistrict_294   Binary   0.0000 Balanced, <0.1
## superdistrict_295   Binary   0.0000 Balanced, <0.1
## superdistrict_296   Binary   0.0000 Balanced, <0.1
## superdistrict_297   Binary   0.0000 Balanced, <0.1
## superdistrict_298   Binary   0.0000 Balanced, <0.1
## superdistrict_299   Binary   0.0000 Balanced, <0.1
## superdistrict_300   Binary   0.0000 Balanced, <0.1
## superdistrict_301   Binary   0.0000 Balanced, <0.1
## superdistrict_302   Binary   0.0000 Balanced, <0.1
## superdistrict_303   Binary   0.0000 Balanced, <0.1
## superdistrict_304   Binary   0.0000 Balanced, <0.1
## superdistrict_305   Binary   0.0000 Balanced, <0.1
## superdistrict_306   Binary   0.0000 Balanced, <0.1
## superdistrict_307   Binary   0.0000 Balanced, <0.1
## superdistrict_308   Binary   0.0000 Balanced, <0.1
## superdistrict_309   Binary   0.0000 Balanced, <0.1
## superdistrict_310   Binary   0.0000 Balanced, <0.1
## superdistrict_311   Binary   0.0000 Balanced, <0.1
## superdistrict_312   Binary   0.0000 Balanced, <0.1
## superdistrict_313   Binary   0.0000 Balanced, <0.1
## superdistrict_314   Binary   0.0000 Balanced, <0.1
## superdistrict_315   Binary   0.0000 Balanced, <0.1
## superdistrict_316   Binary   0.0000 Balanced, <0.1
## superdistrict_317   Binary   0.0000 Balanced, <0.1
## superdistrict_318   Binary   0.0000 Balanced, <0.1
## superdistrict_319   Binary   0.0000 Balanced, <0.1
## superdistrict_320   Binary   0.0000 Balanced, <0.1
## superdistrict_321   Binary   0.0000 Balanced, <0.1
## superdistrict_322   Binary   0.0000 Balanced, <0.1
## superdistrict_323   Binary   0.0000 Balanced, <0.1
## superdistrict_324   Binary   0.0000 Balanced, <0.1
## superdistrict_325   Binary   0.0000 Balanced, <0.1
## superdistrict_326   Binary   0.0000 Balanced, <0.1
## superdistrict_327   Binary   0.0000 Balanced, <0.1
## superdistrict_328   Binary   0.0000 Balanced, <0.1
## superdistrict_329   Binary   0.0000 Balanced, <0.1
```

```
## superdistrict_330   Binary   0.0000 Balanced, <0.1
## superdistrict_331   Binary   0.0000 Balanced, <0.1
## day_1               Binary   0.0000 Balanced, <0.1
## day_2               Binary   0.0000 Balanced, <0.1
## day_3               Binary   0.0000 Balanced, <0.1
## day_4               Binary   0.0000 Balanced, <0.1
## day_5               Binary   0.0000 Balanced, <0.1
## day_6               Binary   0.0000 Balanced, <0.1
## day_7               Binary   0.0000 Balanced, <0.1
## day_8               Binary   0.0000 Balanced, <0.1
## day_9               Binary   0.0000 Balanced, <0.1
## day_10              Binary   0.0000 Balanced, <0.1
## day_11              Binary   0.0000 Balanced, <0.1
## day_12              Binary   0.0000 Balanced, <0.1
## day_13              Binary   0.0000 Balanced, <0.1
## day_14              Binary   0.0000 Balanced, <0.1
## day_15              Binary   0.0000 Balanced, <0.1
## day_16              Binary   0.0000 Balanced, <0.1
## day_17              Binary   0.0000 Balanced, <0.1
## day_18              Binary   0.0000 Balanced, <0.1
## day_19              Binary   0.0000 Balanced, <0.1
## day_20              Binary   0.0000 Balanced, <0.1
## day_21              Binary   0.0000 Balanced, <0.1
## day_22              Binary   0.0000 Balanced, <0.1
## day_23              Binary   0.0000 Balanced, <0.1
## day_24              Binary   0.0000 Balanced, <0.1
## day_25              Binary   0.0000 Balanced, <0.1
## day_26              Binary   0.0000 Balanced, <0.1
## day_27              Binary   0.0000 Balanced, <0.1
## day_28              Binary   0.0000 Balanced, <0.1
## day_29              Binary   0.0000 Balanced, <0.1
## day_30              Binary   0.0000 Balanced, <0.1
## day_31              Binary   0.0000 Balanced, <0.1
## month_1             Binary   0.0000 Balanced, <0.1
## month_2             Binary   0.0000 Balanced, <0.1
## month_3             Binary   0.0000 Balanced, <0.1
## month_4             Binary   0.0000 Balanced, <0.1
## month_5             Binary   0.0000 Balanced, <0.1
## month_6             Binary   0.0000 Balanced, <0.1
## month_7             Binary   0.0000 Balanced, <0.1
## month_8             Binary   0.0000 Balanced, <0.1
## month_9             Binary   0.0000 Balanced, <0.1
## month_10            Binary   0.0000 Balanced, <0.1
## month_11            Binary   0.0000 Balanced, <0.1
## month_12            Binary   0.0000 Balanced, <0.1
## year_2015           Binary   0.0000 Balanced, <0.1
## year_2016           Binary   0.0000 Balanced, <0.1
## year_2017           Binary   0.0000 Balanced, <0.1
## year_2018           Binary   0.0000 Balanced, <0.1
## year_2019           Binary   0.0000 Balanced, <0.1
## year_2020           Binary   0.0000 Balanced, <0.1
## year_2021           Binary   0.0000 Balanced, <0.1
## year_2022           Binary   0.0000 Balanced, <0.1
##
## Balance tally for mean differences
##                    count
## Balanced, <0.1       386
## Not Balanced, >0.1     0
##
## Variable with the greatest mean difference
##  Variable Diff.Adj    M.Threshold
##   COPD_NO   -0.006 Balanced, <0.1
##
## Sample sizes
##          Control Treated
## All       459755  141937
```

```
## Matched     59027   59027
## Unmatched  400728   82910
```

```
#Matched data
load("D:\\Post TB\\TB data\\Results\\Journal submission\\Rcodes\\cc.match2.RData")
#Follow up admission of matched data
load("D:\\Post TB\\TB data\\Results\\Journal submission\\Rcodes\\alladm.RData")
#Disease names in ICD-10 codes
load("D:\\TBsequelae\\update20mar2024\\icd10code.RData")
icd10<-read.csv("D:\\Plos one revise\\icd10.csv")
head(cc.match2)
```

```
##     pid  tran_id    sex marry_status age_y    dateadm  datedsc timeadm timedsc
## 1   273 54177840   Male            1    25 2021-11-24 20211202     128    1526
## 2   352 18396423 Female            2    74 2017-06-03 20170604     840     836
## 5   501 46247008   Male            1    23 2017-02-03 20170206    1521    1100
## 7   593 58178576   Male            3    70 2017-02-25 20170312    1355    1300
## 9   938 23653437   Male            2    71 2020-03-24 20200407    1057    1200
## 10 1134 33847746   Male            2    65 2020-11-15 20201118    1044    1500
##    los dischs discht drg_nhso rw_nhso adjrw_nhso weight mth g_year  pdx sdx1
## 1    0      2      1     4033  3.8822     3.8822      0  11   2565 A162 A188
## 2    0      2      1    10500  0.4244     0.4244      0   6   2560 E110
## 5    0      2      1     4520  0.5449     0.5449     56   2   2560 A150
## 7    0      2      1     7050  1.9548     1.9649      0   2   2560 K810 E119
## 9    0      2      1     4523  1.4948     1.4948     37   3   2563 A162 E876
## 10   0      2      1     4521  0.8752     0.8752     48  11   2564 A160 E119
##    sdx2 sdx3 sdx4 sdx5 sdx6 sdx7 sdx8 sdx9 sdx10 sdx11 sdx12 sdx13 sdx14 sdx15
## 1   D62 J969 K230 R042 Z115
## 2
## 5
## 7
## 9  L891
## 10 E789 J209
##    sdx16 sdx17 sdx18 sdx19 sdx20 proc1 proc2 proc3 proc4 proc5 proc6 proc7
## 1                                 8741  9634  9672  9904
## 2
## 5
## 7                                 5122
## 9
## 10
##    proc8 proc9 proc10 proc11 proc12 proc13 proc14 proc15 proc16 proc17 proc18
## 1
## 2
## 5
## 7
## 9
## 10
##    proc19 proc20 proc21 death_date health_region superdistrict
## 1                                              8           161
## 2                                              9           112
## 5                                             10           119
## 7                                             10           126
## 9                                              1           209
## 10                                             7           173
##                                                          matchdup agegrp year
## 1    Male 25 2021-11-24 20211202 128 1526 0 2 1 A162 3.8822 8 161  25-29 2021
## 2   Female 74 2017-06-03 20170604 840 836 0 2 1 E110 0.4244 9 112  70-74 2017
## 5  Male 23 2017-02-03 20170206 1521 1100 0 2 1 A150 0.5449 10 119  20-24 2017
## 7  Male 70 2017-02-25 20170312 1355 1300 0 2 1 K810 1.9548 10 126  70-74 2017
## 9   Male 71 2020-03-24 20200407 1057 1200 0 2 1 A162 1.4948 1 209  70-74 2020
## 10  Male 65 2020-11-15 20201118 1044 1500 0 2 1 A160 0.8752 7 173  65-69 2020
##    month day       rwgroup                   matchid  group hfdate HF  ihddate
## 1     11  24  (1.49,1e+03] Male 25-29 2021 11 24 161     TB     NA NO       NA
## 2      6   3    (-1,0.545] Female 70-74 2017 6 3 112 Non-TB     NA NO 20170603
## 5      2   3    (-1,0.545]   Male 20-24 2017 2 3 119     TB     NA NO       NA
## 7      2  25  (1.49,1e+03]  Male 70-74 2017 2 25 126 Non-TB     NA NO 20170225
## 9      3  24  (0.875,1.49]  Male 70-74 2020 3 24 209     TB     NA NO       NA
## 10    11  15 (0.545,0.875] Male 65-69 2020 11 15 173     TB     NA NO 20201115
##    IHD hivdate HIV   dmdate DM cadate CA copddate COPD l.age_y l.datedsc
## 1   NO      NA  NO       NA NO     NA NO       NA   NO      25  20211202
## 2  IHD      NA  NO 20170603 DM     NA NO       NA   NO      78  20210912
## 5   NO      NA  NO       NA NO     NA NO       NA   NO      23  20170206
## 7  IHD      NA  NO 20170225 DM     NA NO       NA   NO      75  20220606
## 9   NO      NA  NO       NA NO     NA NO       NA   NO      71  20200407
## 10 IHD      NA  NO 20201115 DM     NA NO       NA   NO      71  20211217
##    l.gyear l.discht l.pdx  distance weights subclass
## 1     2565        0  A162 0.3496986       1        1
## 2     2564        0  E273 0.2267807       1    11373
## 5     2560        0  A150 0.3523575       1        2
```

```
## 7     2565        0  N184 0.2246959       1    29669
## 9     2563        0  A162 0.2913545       1        3
## 10    2565        0  J128 0.2298199       1        4
```

```
cc.match<-cc.match2
casecontrol.alladm<-alladm
cc.match %>% dplyr::rename(dx.dscdate=datedsc) -> cc.match
cc.match %>% dplyr::rename(dx.date=dateadm) -> cc.match
cc.match %>% dplyr::select(pid,group,dx.dscdate) -> cc.group
casecontrol.alladm %>% dplyr::rename(icdadmdate=dateadm)->casecontrol.alladm
casecontrol.alladm<-merge(casecontrol.alladm,cc.group,by="pid")
```

```
setdiff(casecontrol.alladm$pid,cc.match$pid)
```

```
## integer(0)
```

```
setdiff(cc.match$pid,casecontrol.alladm$pid)
```

```
## integer(0)
```

#Table 1 Background characteristics of matched non-TB and TB cohort
#Initial admission

```
#Initial admission
cc.match %>% dplyr::select(group,sex,agegrp,age_y,year,month,DM,HIV,CA,COPD,HF,IHD) -> background
background$month<-factor(background$month)
background %>% group_by(group) %>%
summarise(Q1=quantile(age_y,probs = 0.25,na.rm=TRUE),
                    median=median(age_y,na.rm=TRUE),
          Q3=quantile(age_y, probs = 0.75,na.rm=TRUE))
```

```
## # A tibble: 2 × 4
##   group     Q1 median    Q3
##   <fct>  <dbl>  <int> <dbl>
## 1 Non-TB    48     59    68
## 2 TB        47     58    68
```

```
matchedbac<-table1::table1(~sex+age_y+year+month+DM+HIV+CA+COPD+HF+IHD|group,data=background)
matchedbac
```

	Non-TB (N=59027)	TB (N=59027)	Overall (N=118054)
**sex**			
Female	16201 (27.4%)	16184 (27.4%)	32385 (27.4%)
Male	42826 (72.6%)	42843 (72.6%)	85669 (72.6%)
**age_y**			
Mean (SD)	57.1 (15.2)	57.0 (15.6)	57.0 (15.4)
Median [Min, Max]	59.0 [15.0, 104]	58.0 [15.0, 103]	58.0 [15.0, 104]
**year**			
2015	0 (0%)	0 (0%)	0 (0%)
2016	0 (0%)	0 (0%)	0 (0%)
2017	11802 (20.0%)	11802 (20.0%)	23604 (20.0%)
2018	11387 (19.3%)	11387 (19.3%)	22774 (19.3%)
2019	10942 (18.5%)	10942 (18.5%)	21884 (18.5%)
2020	9778 (16.6%)	9778 (16.6%)	19556 (16.6%)
2021	8891 (15.1%)	8891 (15.1%)	17782 (15.1%)

	Non-TB<br>(N=59027)	TB<br>(N=59027)	Overall<br>(N=118054)
2022	6227 (10.5%)	6227 (10.5%)	12454 (10.5%)
**month**			
1	5469 (9.3%)	5469 (9.3%)	10938 (9.3%)
2	5117 (8.7%)	5117 (8.7%)	10234 (8.7%)
3	5888 (10.0%)	5888 (10.0%)	11776 (10.0%)
4	4970 (8.4%)	4970 (8.4%)	9940 (8.4%)
5	5319 (9.0%)	5319 (9.0%)	10638 (9.0%)
6	5227 (8.9%)	5227 (8.9%)	10454 (8.9%)
7	4853 (8.2%)	4853 (8.2%)	9706 (8.2%)
8	5047 (8.6%)	5047 (8.6%)	10094 (8.6%)
9	4703 (8.0%)	4703 (8.0%)	9406 (8.0%)
10	4425 (7.5%)	4425 (7.5%)	8850 (7.5%)
11	4208 (7.1%)	4208 (7.1%)	8416 (7.1%)
12	3801 (6.4%)	3801 (6.4%)	7602 (6.4%)
**DM**			
DM	12407 (21.0%)	12701 (21.5%)	25108 (21.3%)
NO	46620 (79.0%)	46326 (78.5%)	92946 (78.7%)
**HIV**			
HIV	418 (0.7%)	578 (1.0%)	996 (0.8%)
NO	58609 (99.3%)	58449 (99.0%)	117058 (99.2%)
**CA**			
CA	2809 (4.8%)	2595 (4.4%)	5404 (4.6%)
NO	56218 (95.2%)	56432 (95.6%)	112650 (95.4%)
**COPD**			
COPD	1817 (3.1%)	2169 (3.7%)	3986 (3.4%)
NO	57210 (96.9%)	56858 (96.3%)	114068 (96.6%)
**HF**			
HF	1688 (2.9%)	1902 (3.2%)	3590 (3.0%)
NO	57339 (97.1%)	57125 (96.8%)	114464 (97.0%)
**IHD**			
IHD	16247 (27.5%)	16446 (27.9%)	32693 (27.7%)
NO	42780 (72.5%)	42581 (72.1%)	85361 (72.3%)

```
#Total death and person-years follow-up
cc.match$l.datedsc<-ymd(cc.match$l.datedsc)
cc.match$dx.dscdate<-ymd(cc.match$dx.dscdate)
cc.match %>% filter(l.discht==0) ->cc.match1
cc.match %>% filter(l.discht==1) ->cc.match0


cc.match1$l.datedsc <-"2022-12-30"
cc.match1$l.datedsc<-as.Date(cc.match1$l.datedsc)
cc.match2<-rbind(cc.match1,cc.match0)

class(cc.match$l.datedsc)
```

```
## [1] "Date"
```

```
cc.match2 %>% mutate(duration=l.datedsc-dx.dscdate) -> cc.match2
cc.match2$duration<-as.numeric(cc.match2$duration)
data.pdx.dur<-aggregate(duration~group,cc.match2,sum)
data.pdx.dur %>% mutate(personyear=duration/365) ->data.pdx.dur
td<-aggregate(l.discht~group,cc.match2,sum)
py<-aggregate(personyear~group,data.pdx.dur,sum)
dpy<-merge(td,py,by="group")
dpy %>% dplyr::rename(total.death=l.discht) ->dpy
dpy
```

```
##    group total.death personyear
## 1 Non-TB        3411   187697.1
## 2     TB        6063   179688.9
```

#Univariate analysis #Supplementary Table 1,2 Occurrence of cardiopulmonary sequelae in subsequent admissions of hospitalized patient with TB comparted to those without TB in univariate analysis #Supplementary Table 3 Occurrence of extrapulmonary sequelae in subsequent admissions of hospitalized patient with TB comparted to those without TB in univariate analysis

```
#Identifying cardiopulmonary sequalae in primary and secondary diagnosis
casecontrol.alladm$icdadmdate<-ymd(casecontrol.alladm$icdadmdate)
casecontrol.alladm$dx.dscdate<-ymd(casecontrol.alladm$dx.dscdate)
#Duration of occurrence of sequelae in subsequent admissions from discharge date of index admission
#dateadm is admission date for post-TB diseases
#dx.dscdate is discharge date of initial TB and non-TB case
casecontrol.alladm %>% mutate(duration=icdadmdate-dx.dscdate) ->casecontrol.alladm
casecontrol.alladm %>% dplyr::rename(sdx0=pdx) -> match.all
match.all %>% dplyr::select(pid,dx.dscdate,icdadmdate,duration,19:39) ->match.all

match.all %>%
  pivot_longer(
    cols = starts_with("sdx"),
    values_to = "Disease") ->match.all.long

#Convert ICD-10 code into three digits
match.all.long$duration<-as.numeric(match.all.long$duration)
match.all.long$pdx.2<-match.all.long$Disease
match.all.long$strno <-nchar(match.all.long$pdx.2)

match.all.long %>% filter(strno<=3) ->match.all0
match.all.long %>% filter(strno==4) ->match.all1
match.all.long %>% filter(strno>4) ->match.all2

match.all1$pdx.2<-substr(match.all1$pdx.2,1,nchar(match.all1$pdx.2)-1)
match.all2$pdx.2<-substr(match.all2$pdx.2,1,nchar(match.all2$pdx.2)-2)

match.all.long<-rbind(match.all1,match.all2,match.all0)
names(match.all.long)
```

```
## [1] "pid"        "dx.dscdate" "icdadmdate" "duration"   "name"
## [6] "Disease"    "pdx.2"      "strno"
```

```
match.all.long %>% dplyr::select(pid,dx.dscdate,icdadmdate,duration,pdx.2) -> match.all6m

match.all6m %>% filter(duration>0) %>% head(10) #pid with their subsequent sequel in their follow-up admissions (duration)
```

```
## # A tibble: 10 × 5
##      pid dx.dscdate icdadmdate duration pdx.2
##    <int> <date>     <date>        <dbl> <chr>
##  1   352 2017-06-04 2021-09-09     1558 E27
##  2   352 2017-06-04 2021-09-09     1558 D64
##  3   352 2017-06-04 2021-09-09     1558 E11
##  4   352 2017-06-04 2021-09-09     1558 E78
##  5   352 2017-06-04 2021-09-09     1558 E87
##  6   352 2017-06-04 2021-09-09     1558 Z11
##  7   352 2017-06-04 2021-08-30     1548 E11
##  8   352 2017-06-04 2021-08-30     1548 E87
##  9   352 2017-06-04 2021-08-30     1548 Z11
## 10   593 2017-03-12 2022-05-30     1905 N18
```

```
match.all6m %>% dplyr::select(pid,icdadmdate,duration,pdx.2)->match.all6m

match.all6m %>% count(pdx.2) %>% arrange(desc(n)) ->topdis

topdis %>% slice(-1) ->topdis
#Filter ICD-10 codes of J|I for cardio-pulmonary sequeale
#Filter ICD-10 codes of E|G|K|M|N for endocrine (E00-E35), central nervous system (G00-G99), digestive system (K00-K95), mus
culoskeletal system (M00-M99), and genitourinary system (N00-N99)
#topdis %>% filter(grepl('N', pdx.2)) -> topdis.top
topdis %>% filter(grepl('I32|J93|J85|J47|J86|J90|J15|J96|E22|G01|K75', pdx.2)) -> topdis.top
#cardiopulmonary sequalae
topdis.top %>% filter(n>10) -> topdis.top
```

```
#Merge pid of occurrence of each sequelae to their respective case and control
dfList <- list()
for (i in c(1:nrow(topdis.top))) {
  match.all6m %>% filter(pdx.2==topdis.top$pdx.2[i]) ->match.allpdx2
  match.allpdx2  %>% group_by(pid)  %>% arrange (icdadmdate) %>% slice_head(n=1) ->match.allpdx2
  disease<-merge(cc.match,match.allpdx2,all.x=TRUE,by="pid")
#Filter out the matched pairs with prevalence a ICD disease
  prev_id <- unique(disease$subclass[disease$duration <= 0])
  prevelancedi_remove <- disease[!disease$subclass %in% prev_id, ]
  prevelancedi_remove %>% mutate_at(vars(-c(1:93)), ~replace(., is.na(.), "No")) -> disease
  disease$pdx.2<-factor(disease$pdx.2)
  disease$group<-factor(disease$group)
  disease$l.datedsc<-ymd(disease$l.datedsc)
  disease$l.datedsc<-as.character(disease$l.datedsc)
  disease$icdadmdate<-as.character(disease$icdadmdate)
  #If there is occurrence of sequelae, admission date that occur it will be event date
  disease %>% filter(!pdx.2=="No") ->disease1

  disease %>% filter(pdx.2=="No") ->disease0

  #If there is no occurrence of sequelae and the patients is death, last #discharge date will be used as the death date.

  disease0 %>% filter(l.discht=="1") ->disease01
  disease0 %>% filter(l.discht=="0") ->disease00

disease01 %>% dplyr::mutate(icdadmdate=if_else(is.na(icdadmdate),coalesce(icdadmdate, l.datedsc),icdadmdate)) -> disease01

  #If there is no occurrence of sequelae, the patients will be follow-up until  #"2022-12-30"
disease00$icdadmdate [is.na(disease00$icdadmdate)]<-"2022-12-30"

 mdata.all<-rbind(disease1,disease01,disease00)


mdata.all$icdadmdate<- ymd(mdata.all$icdadmdate)
mdata.all$dx.dscdate<- ymd(mdata.all$dx.dscdate)

mdata.all %>% mutate(duration=icdadmdate-dx.dscdate) ->mdata.all
mdata.all$duration[mdata.all$duration<0]<-0
dfList[[i]] <-mdata.all
}
```

```
table(dfList[[1]]$group,dfList[[1]]$pdx.2) #Occurance of sequelae
```

```
##
##            J96    No
##   Non-TB  2172 46480
##   TB      2466 46186
```

```
table(dfList[[2]]$group,dfList[[2]]$pdx.2) #Occurance of sequelae
```

```
##
##            J15    No
##   Non-TB  1168 51142
##   TB      1513 50797
```

#Univariate analysis of association between TB and sequelae using stratified log rank test

```
obs_results<-NULL
obs_results <- data.frame()
a<-NULL
data.pdx.sexdur1<-NULL
data.pdx.sexdur1<-data.frame()
data.pdx.sexdur2<-NULL

data.pdx.sexdur2<-data.frame()
options("scipen"=100, "digits"=4)
for (i in c(1:max(seq(dfList)))){
 data.pdx<- dfList[[i]]
  data.pdx$duration<-as.numeric(data.pdx$duration)
  data.pdx$duration<-data.pdx$duration+1
  twobytwo<-table(data.pdx$pdx.2,data.pdx$group,useNA = "always")
  data.pdx$event<-data.pdx$pdx.2
  data.pdx$event<-factor(data.pdx$event)
  data.pdx$event<-as.numeric(data.pdx$event)
  data.pdx$event<-ifelse(data.pdx$event==1,1,0)
  data.pdx$group<-factor(data.pdx$group)
  data.pdx$agep<-cut(data.pdx$age_y,breaks = c(0,40,60,80,120))

   #
#Difference in survival distribution of occurrence of sequelae among TB and non-TB using stratified log-rank test

  fit <- survdiff(Surv(duration,event) ~ group + strata(subclass), data = data.pdx)

# p-value of stratified log rank test
logrpval<-fit$pvalue
logrpval<-round(logrpval,4)

#Count event and non-event of sequelae
data.pdx %>% group_by(group) %>% dplyr::count(event) %>% filter(event==1)->data.pdx.event
  data.pdx %>% group_by(group) %>% dplyr::count(event) %>% filter(event==0)->data.pdx.noevent
  #Person-years of follow-up for occurrence of sequelae
  data.pdx.dur<-aggregate(duration~group,data.pdx,sum)
  data.pdx.dur %>% mutate(personyear=duration/365) ->data.pdx.dur
  data.pdx.sexdur<-merge(data.pdx.noevent,data.pdx.event,by="group")
  data.pdx.sexdur<-merge(data.pdx.sexdur,data.pdx.dur,by="group")
  #Calculate incidence rate per 100,000 person-years
  data.pdx.sexdur %>% mutate(incidence=n.y/personyear*100000) ->data.pdx.sexdur
  data.pdx.sexdur %>% dplyr::rename(Noevent=n.x) ->data.pdx.sexdur
  data.pdx.sexdur %>% dplyr::rename(Event=n.y) ->data.pdx.sexdur

  data.pdx.sexdur %>% dplyr::select(group,Noevent,Event,personyear,incidence) ->data.pdx.sexdur
  data.pdx.sexdur$personyear<-round(data.pdx.sexdur$personyear,0)
  data.pdx.sexdur$incidence<-round(data.pdx.sexdur$incidence,0)
  data.pdx.sexdur<-data.frame(data.pdx.sexdur)
  data.pdx.sexdur1[i,1:5]<-data.pdx.sexdur[1,1:5]
  data.pdx.sexdur2[i,1:5]<-data.pdx.sexdur[2,1:5]
  data.pdx.sexdur1$Disease[i]<-rownames(twobytwo)[[1]]
  data.pdx.sexdur2$Disease[i]<-rownames(twobytwo)[[1]]
  options("scipen"=100, "digits"=2)
    obs_results[i,1] <- rownames(twobytwo)[[1]]
    obs_results[i,2] <-round(logrpval,3)
  colnames(obs_results) <-c("Disease","stratified log-rank test_pvalue")
  }
```

```
obs_results
```

```
##    Disease stratified log-rank test_pvalue
## 1      J96                           0.000
## 2      J15                           0.000
## 3      J90                           0.000
## 4      K75                           0.000
## 5      J93                           0.000
## 6      J47                           0.000
## 7      E22                           0.000
## 8      J86                           0.001
## 9      J85                           0.000
## 10     G01                           0.000
## 11     I32                           0.001
```

```
obs_results %>% arrange(desc("stratified log-rank test_pvalue")) -> obs_results1
head(obs_results1)
```

```
##   Disease stratified log-rank test_pvalue
## 1     J96                               0
## 2     J15                               0
## 3     J90                               0
## 4     K75                               0
## 5     J93                               0
## 6     J47                               0
```

#95% CI of incidence per 100 000 person-years

```
#Event of sequelae, non-event, person-years of follow up and incidence per 100,000 person-years for each sequelae in TB case
s and non-TB control
data.pdx.sexdur.obs12<-rbind(data.pdx.sexdur1,data.pdx.sexdur2)
data.pdx.sexdur.obs12 %>% dplyr::select(group,Noevent,Event,personyear,incidence,Disease) ->data.pdx.sexdur.obs12
head(data.pdx.sexdur.obs12)
```

```
##    group Noevent Event personyear incidence Disease
## 1 Non-TB   46480  2172     156430      1388     J96
## 2 Non-TB   51142  1168     166168       703     J15
## 3 Non-TB   55659   400     178221       224     J90
## 4 Non-TB   56655   323     181048       178     K75
## 5 Non-TB   57745    82     184240        45     J93
## 6 Non-TB   58251    81     185628        44     J47
```

```
#Calculate 95% CI of incidence of sequelae
data.pdx.sexdur.obs12 %>% group_split(Disease) ->  data.pdx.sexdur.obs12

peryr95_results<-NULL
for (i in c(1:length(data.pdx.sexdur.obs12))){
  data.peryr95<-data.pdx.sexdur.obs12[[i]]

  tmp <- as.matrix(cbind(data.peryr95$Event,data.peryr95$personyear))
  per95<-epi.conf(tmp, ctype = "inc.rate", method = "exact", N = 1000, design = 1,

                  conf.level = 0.95) * 100000
  per95$est<-round(per95$est,0)
  per95$lower<-round(per95$lower,0)
  per95$upper<-round(per95$upper,0)
  per95res<-paste0(per95$est,"(",per95$lower,"-",per95$upper,")")
  data.peryr95<-cbind(data.peryr95,per95res)
  peryr95_results[[i]]<-data.peryr95
}


peryr95_results %>% bind_rows() -> data.pdx.sexdur.obs12
head(data.pdx.sexdur.obs12)
```

```
##    group Noevent Event personyear incidence Disease    per95res
## 1 Non-TB   58202    78     185713        42     E22   42(33-52)
## 2     TB   58080   200     177596       113     E22 113(98-129)
## 3 Non-TB   58890     3     187514         2     G01      2(0-5)
## 4     TB   58838    55     179431        31     G01   31(23-40)
## 5 Non-TB   58967     5     187672         3     I32      3(1-6)
## 6     TB   58949    23     179623        13     I32    13(8-19)
```

```
data.pdx.sexdur.obs12 %>% filter(is.na(personyear))
```

```
## [1] group      Noevent    Event      personyear incidence  Disease    per95res
## <0 rows> (or 0-length row.names)
```

```
data.pdx.sexdur.obs12 %>% filter(!Disease=="No") -> data.pdx.sexdur.obs12
```

```
#Wide form
data.pdx.sexdur.obs12 %>%
  tidyr::pivot_wider(names_from = group,
                     values_from = c(Noevent,Event,personyear,incidence,per95res)) ->data.pdx.sexdur.obs122
head(data.pdx.sexdur.obs122)
```

```
## # A tibble: 6 × 11
##   Disease `Noevent_Non-TB` Noevent_TB `Event_Non-TB` Event_TB
##   <chr>              <int>      <int>          <int>    <int>
## 1 E22                58202      58080             78      200
## 2 G01                58890      58838              3       55
## 3 I32                58967      58949              5       23
## 4 J15                51142      50797           1168     1513
## 5 J47                58251      58078             81      254
## 6 J85                58704      58660             38       82
## # ℹ 6 more variables: `personyear_Non-TB` <dbl>, personyear_TB <dbl>,
## #   `incidence_Non-TB` <dbl>, incidence_TB <dbl>, `per95res_Non-TB` <chr>,
## #   per95res_TB <chr>
```

```
cardiorepiratorysequeale<-merge(data.pdx.sexdur.obs122,obs_results,by="Disease")
names(cardiorepiratorysequeale)
```

```
##  [1] "Disease"                          "Noevent_Non-TB"
##  [3] "Noevent_TB"                       "Event_Non-TB"
##  [5] "Event_TB"                         "personyear_Non-TB"
##  [7] "personyear_TB"                    "incidence_Non-TB"
##  [9] "incidence_TB"                     "per95res_Non-TB"
## [11] "per95res_TB"                      "stratified log-rank test_pvalue"
```

```
cardiorepiratorysequeale %>% select(Disease,Event_TB,personyear_TB,per95res_TB,"Event_Non-TB","personyear_Non-TB","per95res_
Non-TB","stratified log-rank test_pvalue") ->cardiorepiratorysequeale
head(cardiorepiratorysequeale)
```

```
##   Disease Event_TB personyear_TB   per95res_TB Event_Non-TB personyear_Non-TB
## 1     E22      200        177596   113(98-129)           78            185713
## 2     G01       55        179431     31(23-40)            3            187514
## 3     I32       23        179623      13(8-19)            5            187672
## 4     J15     1513        158812 953(905-1002)         1168            166168
## 5     J47      254        177304  143(126-162)           81            185628
## 6     J85       82        178856     46(36-57)           38            186983
##   per95res_Non-TB stratified log-rank test_pvalue
## 1       42(33-52)                           0.000
## 2          2(0-5)                           0.000
## 3          3(1-6)                           0.001
## 4    703(663-744)                           0.000
## 5       44(35-54)                           0.000
## 6       20(14-28)                           0.000
```

```
# ICD-10 disease with disease names
cardiorepiratorysequeale %>% group_split(Disease) ->cardiorepiratorysequeale

cardiorepiratorysequeale %>% bind_rows() -> cardiorepiratorysequeale



cardiorepiratorysequeale %>% dplyr::rename("ICD.10"="Disease")-> cardiorepiratorysequeale

cardiorepiratorysequeale<-merge(cardiorepiratorysequeale,icd10code,all.x=TRUE,by="ICD.10")
head(cardiorepiratorysequeale)
```

```
##   ICD.10 Event_TB personyear_TB   per95res_TB Event_Non-TB personyear_Non-TB
## 1    E22      200        177596   113(98-129)           78            185713
## 2    G01       55        179431     31(23-40)            3            187514
## 3    I32       23        179623      13(8-19)            5            187672
## 4    J15     1513        158812 953(905-1002)         1168            166168
## 5    J47      254        177304  143(126-162)           81            185628
## 6    J85       82        178856     46(36-57)           38            186983
##   per95res_Non-TB stratified log-rank test_pvalue
## 1       42(33-52)                           0.000
## 2          2(0-5)                           0.000
## 3          3(1-6)                           0.001
## 4    703(663-744)                           0.000
## 5       44(35-54)                           0.000
## 6       20(14-28)                           0.000
##                                                major
## 1                         Hyperfunction of pituitary gland
## 2 Meningitis in bacterial diseases classified elsewhere
## 3         Pericarditis in diseases classified elsewhere
## 4         Bacterial pneumonia, not elsewhere classified
## 5                                        Bronchiectasis
## 6                          Abscess of lung and mediastinum
```

```
#write.csv(cardiorepiratorysequeale,"D:\\Post TB\\TB #data\\Results\\Journal submission\\Genttoursequeale229.9.2025.csv")
```

```
#The higher incidence of sequelae in TB and the significant difference in survival distribution between TB and non-TB cases were used for further analysis
#I32, J43
topdis.top$id<-seq(1:nrow(topdis.top))

#2 (J96)
#79 (I32), #20 (J93) #50 (J85) #27 (J47)   #46 (J86)  #11 (J90) #4 (J15)
#14(E22)#19(G01)#6(K75)
```

Table 2 Risk of occurrence of sequelae and death in hospitalized patients with TB and non-TB in Thailand in multivariate analysis

#Multivariate analysis of association between TB and sequeale using a robust Cox regression with a robust variance sandwich estimator

```
#dfList[[79]](ICD-10 I32 (Pericarditis) in TB and non TB), n=117944
#dfList[[20]] (ICD-10 J93 (Pneumothorax) in TB and non TB), n=115654
#dfList[[50]] (ICD-10 J85 (Abscess of lung and mediastinum) in TB and non TB),n=117484
#dfList[[27]] (ICD-10 J47 (Bronchiectasis) in TB and non TB),n=116664
#dfList[[46]] (ICD-10 J86 (Pyothorax) in TB and non TB), n= 117458
#dfList[[11]] (ICD-10 J90 (Pleural effusion) in TB and non TB), n=112118
#dfList[[4]] (ICD-10 J15 (Pneumonia) in TB and non TB),n=104620
#dfList[[2]] (ICD-10 J96 (Respiratory failure) in TB and non TB), n=97304


#A robust Cox regression with a robust variance sandwich estimator
data.pdx<-dfList[[2]]
  data.pdx$duration<-as.numeric(data.pdx$duration)
  data.pdx$duration<-data.pdx$duration+1
  twobytwo<-table(data.pdx$pdx.2,data.pdx$group,useNA = "always")
  twobytwo
```

```
##
##        Non-TB    TB  <NA>
##   J15    1168  1513     0
##   No    51142 50797     0
##   <NA>      0     0     0
```

```
  data.pdx$event<-data.pdx$pdx.2
  data.pdx$event<-factor(data.pdx$event)
  data.pdx$event<-as.numeric(data.pdx$event)
  data.pdx$event<-ifelse(data.pdx$event==1,1,0)
  data.pdx$group<-factor(data.pdx$group)
  data.pdx$g_year<-factor(data.pdx$g_year)
  data.pdx$agep<-cut(data.pdx$age_y,breaks =c(0,40,60,80,120))

fit2 <- coxphw(Surv(duration, event) ~ group+agep+sex+COPD+HIV+DM+CA+HF+IHD, data = data.pdx,robust=TRUE,template = "PH")
summary(fit2)
```

```
## coxphw(formula = Surv(duration, event) ~ group + agep + sex +
##     COPD + HIV + DM + CA + HF + IHD, data = data.pdx, template = "PH",
##     robust = TRUE)
##
## Model fitted by unweighted estimation (PH template)
##
##                 coef se(coef) exp(coef) lower 0.95 upper 0.95      z
## groupTB        0.281    0.039      1.32       1.23       1.43   7.19
## agep(40,60]    0.026    0.067      1.03       0.90       1.17   0.39
## agep(60,80]    0.110    0.067      1.12       0.98       1.27   1.65
## agep(80,120]   0.092    0.109      1.10       0.88       1.36   0.84
## sexMale        0.147    0.045      1.16       1.06       1.26   3.31
## COPDNO        -0.972    0.068      0.38       0.33       0.43 -14.30
## HIVNO         -0.442    0.141      0.64       0.49       0.85  -3.13
## DMNO          -0.217    0.071      0.81       0.70       0.93  -3.05
## CANO          -0.370    0.082      0.69       0.59       0.81  -4.49
## HFNO          -0.833    0.068      0.43       0.38       0.50 -12.18
## IHDNO         -0.614    0.081      0.54       0.46       0.64  -7.54
##                              p
## groupTB      0.000000000000627
## agep(40,60]  0.699386656201166
## agep(60,80]  0.098365309144853
## agep(80,120] 0.401341424764497
## sexMale      0.000940369084404
## COPDNO       0.000000000000000
## HIVNO        0.001742923586960
## DMNO         0.002317506390305
## CANO         0.000007083578485
## HFNO         0.000000000000000
## IHDNO        0.000000000000047
##
## Wald Chi-square = 1630 on 11  df  p = 0  n = 104620
##
## Covariance-Matrix:
##                  groupTB agep(40,60] agep(60,80] agep(80,120]     sexMale
## groupTB        0.0015203    0.000032    0.000017   -0.0001189  0.00000692
## agep(40,60]    0.0000323    0.004425    0.003486    0.0034480 -0.00012739
## agep(60,80]    0.0000172    0.003486    0.004430    0.0036627  0.00012161
## agep(80,120] -0.0001189    0.003448    0.003663    0.0119320  0.00028079
## sexMale        0.0000069   -0.000127    0.000122    0.0002808  0.00198349
## COPDNO         0.0001115   -0.000036    0.000215    0.0005972  0.00030014
## HIVNO          0.0000562   -0.000228   -0.000491   -0.0004706 -0.00001655
## DMNO           0.0000773    0.000185   -0.000028   -0.0006199 -0.00024706
## CANO          -0.0000349    0.000027    0.000012   -0.0003911 -0.00000077
## HFNO          -0.0000662   -0.000061   -0.000021    0.0000021 -0.00000033
## IHDNO          0.0001345    0.000388    0.000714    0.0011403 -0.00001284
##                  COPDNO     HIVNO      DMNO        CANO        HFNO     IHDNO
## groupTB        0.000111  0.000056  0.000077 -0.00003485 -0.00006620  0.000134
## agep(40,60]   -0.000036 -0.000228  0.000185  0.00002676 -0.00006132  0.000388
## agep(60,80]    0.000215 -0.000491 -0.000028  0.00001181 -0.00002141  0.000714
## agep(80,120]   0.000597 -0.000471 -0.000620 -0.00039106  0.00000212  0.001140
## sexMale        0.000300 -0.000017 -0.000247 -0.00000077 -0.00000033 -0.000013
## COPDNO         0.004622  0.000763  0.001818  0.00133362 -0.00010970 -0.002645
## HIVNO          0.000763  0.019966  0.000793  0.00094756  0.00083815 -0.001904
## DMNO           0.001818  0.000793  0.005060  0.00268322  0.00055387 -0.004680
## CANO           0.001334  0.000948  0.002683  0.00677996  0.00119357 -0.003645
## HFNO          -0.000110  0.000838  0.000554  0.00119357  0.00467932 -0.001509
## IHDNO         -0.002645 -0.001904 -0.004680 -0.00364537 -0.00150884  0.006625
##
## Generalized concordance probability:   Estimates may be biased!
##              concordance prob. lower 0.95 upper 0.95
## groupTB                   0.57       0.55       0.59
## agep(40,60]               0.51       0.47       0.54
## agep(60,80]               0.53       0.49       0.56
## agep(80,120]              0.52       0.47       0.58
## sexMale                   0.54       0.52       0.56
## COPDNO                    0.27       0.25       0.30
```

```
## HIVNO                       0.39       0.33       0.46
## DMNO                        0.45       0.41       0.48
## CANO                        0.41       0.37       0.45
## HFNO                        0.30       0.28       0.33
## IHDNO                       0.35       0.32       0.39
```

```
library(EValue)
```

```
## Warning: package 'EValue' was built under R version 4.4.3
```

```
evalue.po<-evalues.HR(2.0,1.8,2.4, rare = 1)
evalue.po
```

```
##          point lower upper
## RR         2.0   1.8   2.4
## E-values   3.4   3.0    NA
```

###Death

```
tmp <- as.matrix(cbind(dpy$total.death,dpy$personyear))
tmp
```

```
##      [,1]   [,2]
## [1,] 3411 187697
## [2,] 6063 179689
```

```
per95<-epi.conf(tmp, ctype = "inc.rate", method = "exact", N = 1000, design = 1,

                  conf.level = 0.95) * 100000
per95
```

```
##    est lower upper
## 1 1817  1757  1879
## 2 3374  3290  3460
```

#Association between TB and death

```
cc.match$event<-cc.match$l.discht
table(cc.match$event,cc.match$group)
```

```
##    
##     Non-TB    TB
##   0  55616 52964
##   1   3411  6063
```

```
cc.match$event<-factor(cc.match$event)
cc.match$event<-as.numeric(cc.match$event)
#cc.match$event<-ifelse(cc.match$event==2,1,0)
cc.match$event<-factor(cc.match$event)
cc.match$group<-factor(cc.match$group)
cc.match$group<-relevel(cc.match$group,ref="Non-TB")
class(cc.match$l.datedsc)
```

```
## [1] "Date"
```

```
cc.match$l.datedsc<-ymd(cc.match$l.datedsc)
class(cc.match$dx.date)
```

```
## [1] "Date"
```

```
table(cc.match$group)
```

```
##
## Non-TB     TB
##  59027  59027
```

```
cc.match %>% mutate(duration=l.datedsc-dx.date) -> cc.match
cc.match$duration<-as.numeric(cc.match$duration)
cc.match$duration[cc.match$duration==0]<-1
cc.match$agep<-cut(cc.match$age_y,breaks = c(0,40,60,80,120))


fit <- coxphw(Surv(duration, event) ~ group+agep+sex+COPD+HIV+DM+CA+HF+IHD, data = cc.match,robust=TRUE,template = "PH")
summary(fit)
```

```
## coxphw(formula = Surv(duration, event) ~ group + agep + sex +
##     COPD + HIV + DM + CA + HF + IHD, data = cc.match, template = "PH",
##     robust = TRUE)
##
## Model fitted by unweighted estimation (PH template)
##
##                  coef se(coef) exp(coef) lower 0.95 upper 0.95     z
## groupTB        0.6707    0.021      1.96       1.88       2.04 31.58
## agep(40,60]    0.2740    0.037      1.32       1.22       1.41  7.39
## agep(60,80]    0.4522    0.037      1.57       1.46       1.69 12.35
## agep(80,120]   0.7623    0.052      2.14       1.93       2.37 14.59
## sexMale        0.1502    0.024      1.16       1.11       1.22  6.30
## COPDNO         0.1611    0.053      1.17       1.06       1.30  3.07
## HIVNO         -0.0855    0.090      0.92       0.77       1.10 -0.95
## DMNO           0.2935    0.046      1.34       1.23       1.47  6.45
## CANO          -0.2707    0.049      0.76       0.69       0.84 -5.53
## HFNO          -0.2414    0.047      0.79       0.72       0.86 -5.09
## IHDNO          0.0067    0.051      1.01       0.91       1.11  0.13
##                              p
## groupTB      0.00000000000000
## agep(40,60]  0.00000000000014
## agep(60,80]  0.00000000000000
## agep(80,120] 0.00000000000000
## sexMale      0.00000000030247
## COPDNO       0.00215707595309
## HIVNO        0.34388260771633
## DMNO         0.00000000011408
## CANO         0.00000003233079
## HFNO         0.00000036247614
## IHDNO        0.89615396360776
##
## Wald Chi-square = 1559 on 11  df  p = 0  n = 118054
##
## Covariance-Matrix:
##                  groupTB agep(40,60] agep(60,80] agep(80,120]   sexMale
## groupTB        0.0004510   0.0000111   0.0000169    -0.000027 -0.000015
## agep(40,60]    0.0000111   0.0013730   0.0010939     0.001084 -0.000013
## agep(60,80]    0.0000169   0.0010939   0.0013405     0.001108  0.000053
## agep(80,120] -0.0000274   0.0010835   0.0011077     0.002730  0.000120
## sexMale       -0.0000150  -0.0000129   0.0000532     0.000120  0.000569
## COPDNO         0.0000104  -0.0000118   0.0000493     0.000195  0.000102
## HIVNO          0.0000599   0.0000378  -0.0000327     0.000051  0.000037
## DMNO           0.0000287   0.0000308   0.0000058    -0.000048 -0.000026
## CANO           0.0000537   0.0000022   0.0000100     0.000019  0.000024
## HFNO           0.0000375   0.0000033   0.0000305     0.000076  0.000026
## IHDNO         -0.0000044   0.0000519   0.0000880     0.000055 -0.000035
##                  COPDNO     HIVNO       DMNO       CANO       HFNO      IHDNO
## groupTB        0.000010  0.000060  0.0000287  0.0000537  0.0000375 -0.0000044
## agep(40,60]   -0.000012  0.000038  0.0000308  0.0000022  0.0000033  0.0000519
## agep(60,80]    0.000049 -0.000033  0.0000058  0.0000100  0.0000305  0.0000880
## agep(80,120]   0.000195  0.000051 -0.0000477  0.0000190  0.0000755  0.0000550
## sexMale        0.000102  0.000037 -0.0000255  0.0000237  0.0000258 -0.0000346
## COPDNO         0.002758  0.000506  0.0009324  0.0007766  0.0003010 -0.0012934
## HIVNO          0.000506  0.008167  0.0005975  0.0007064  0.0005613 -0.0010539
## DMNO           0.000932  0.000597  0.0020723  0.0012355  0.0006011 -0.0019710
## CANO           0.000777  0.000706  0.0012355  0.0023980  0.0007921 -0.0016922
## HFNO           0.000301  0.000561  0.0006011  0.0007921  0.0022515 -0.0010527
## IHDNO         -0.001293 -0.001054 -0.0019710 -0.0016922 -0.0010527  0.0026126
##
## Generalized concordance probability:   Estimates may be biased!
##              concordance prob. lower 0.95 upper 0.95
## groupTB                   0.66       0.65       0.67
## agep(40,60]               0.57       0.55       0.59
## agep(60,80]               0.61       0.59       0.63
## agep(80,120]              0.68       0.66       0.70
## sexMale                   0.54       0.53       0.55
## COPDNO                    0.54       0.51       0.57
```

```
## HIVNO                        0.48       0.43       0.52
## DMNO                         0.57       0.55       0.59
## CANO                         0.43       0.41       0.46
## HFNO                         0.44       0.42       0.46
## IHDNO                        0.50       0.48       0.53
```

```
library(EValue)
evalue.po<-evalues.HR(1.9,1.8,2.0, rare = 1)
evalue.po
```

```
##            point lower upper
## RR           1.9   1.8     2
## E-values     3.2   3.0    NA
```

#Table 3 Comparison of average age standardized death rate and years of life lost (YLL) per 100000 per year among the hospitalized patients with TB and non-TB control

```
cc.match$l.age_y[cc.match$l.age_y<15]<-15
cc.match.age<-cc.match
age_groups(cc.match.age$l.age_y, c(5,10,15,20,25,30,35,40,45,50,55,60,65,70,75,80,85)) ->cc.match.age$agegrp
SLE<-c(
89.41,
84.52,
79.53,
74.54,
69.57,
64.6,
59.63,
54.67,
49.73,
44.81,
39.92,
35.07,
30.25,
25.49,
20.77,
16.43,
12.51,
7.6)


stdpop<-c(
  0.0886,
  0.0869,
  0.086,
  0.0847,
  0.0822,
  0.0793,
  0.0761,
  0.0715,
  0.0659,
  0.0604,
  0.0537,
  0.0455,
  0.0372,
  0.0296,
  0.0221,
  0.0152,
  0.0091,
  0.0063)


agegp<-table(cc.match.age$agegrp)
agegp<-data.frame(agegp)
sleage<-cbind(agegp,SLE,stdpop)
sleage %>% dplyr::select(Var1,SLE,stdpop) ->sleage
colnames(sleage)<-c("agegrp","SLE","stdpop") #SLE=Standard life expectancy
sleage
```

```
##    agegrp  SLE stdpop
## 1     0-4 89.4 0.0886
## 2     5-9 84.5 0.0869
## 3   10-14 79.5 0.0860
## 4   15-19 74.5 0.0847
## 5   20-24 69.6 0.0822
## 6   25-29 64.6 0.0793
## 7   30-34 59.6 0.0761
## 8   35-39 54.7 0.0715
## 9   40-44 49.7 0.0659
## 10  45-49 44.8 0.0604
## 11  50-54 39.9 0.0537
## 12  55-59 35.1 0.0455
## 13  60-64 30.2 0.0372
## 14  65-69 25.5 0.0296
## 15  70-74 20.8 0.0221
## 16  75-79 16.4 0.0152
## 17  80-84 12.5 0.0091
## 18    85+  7.6 0.0063
```

```
cc.match.age %>% filter(l.discht=="1") ->cc.match.all.death
cc.match.all.death$dx.date<-year(cc.match.all.death$dx.date)
ccmortal<-aggregate(l.discht~group+agegrp+dx.date,data=cc.match.all.death,sum)
ccmortal.ave<-aggregate(l.discht~group+agegrp,ccmortal,mean)
yll.ave<-merge(ccmortal.ave,sleage,by="agegrp")
yll.ave$l.discht<-round(yll.ave$l.discht, 0)

yll.ave %>% select(agegrp,group,stdpop,SLE,l.discht) ->yll.ave
yll.ave %>% group_split(group) -> yll.ave
yll.ave %>% bind_rows() -> yll.ave

yll.ave %>% mutate(asdr=stdpop*l.discht) -> yll.ave #Age standardized death rate

yll.ave %>% mutate(YLL= SLE*l.discht) ->yll.ave #YLL
yll.ave %>% mutate(YLL.100000=YLL/(stdpop*100000)) ->yll.ave #YLL per 100,000 population
yll.ave %>% dplyr::rename(death=l.discht) -> yll.ave
yll.ave$asdr <- round(yll.ave$asdr, 1)
yll.ave$YLL <- round(yll.ave$YLL, 1)
yll.ave$YLL.100000 <- round(yll.ave$YLL.100000, 3)
```

```
#write.csv(yll.ave,file="D:\\Plos one revise\\yll.ave.csv")
```

```
aggregate(cbind(death,asdr,YLL,YLL.100000)~group,yll.ave,sum)
```

```
##    group death asdr   YLL YLL.100000
## 1 Non-TB   570   21 16893        5.1
## 2     TB  1009   39 31934        9.0
```

#Table 4 Comparison of total years of life lost (YLL) per 100000 per year in hospitalized patients with TB and non-TB controls across both sexes in Thailand for the periods of 2017 and 2022

```
death.data<-merge(ccmortal,sleage,by="agegrp")
death.data
```

```
##     agegrp  group dx.date l.discht  SLE stdpop
## 1    15-19 Non-TB    2017        4 74.5 0.0847
## 2    15-19     TB    2017        9 74.5 0.0847
## 3    15-19 Non-TB    2022        1 74.5 0.0847
## 4    15-19     TB    2022        7 74.5 0.0847
## 5    15-19 Non-TB    2018        5 74.5 0.0847
## 6    15-19     TB    2018        7 74.5 0.0847
## 7    15-19 Non-TB    2020        6 74.5 0.0847
## 8    15-19 Non-TB    2019        8 74.5 0.0847
## 9    15-19     TB    2019        9 74.5 0.0847
## 10   15-19 Non-TB    2021        2 74.5 0.0847
## 11   15-19     TB    2021        5 74.5 0.0847
## 12   15-19     TB    2020       19 74.5 0.0847
## 13   20-24     TB    2020       15 69.6 0.0822
## 14   20-24 Non-TB    2019        2 69.6 0.0822
## 15   20-24 Non-TB    2018        8 69.6 0.0822
## 16   20-24 Non-TB    2017        3 69.6 0.0822
## 17   20-24     TB    2017        7 69.6 0.0822
## 18   20-24 Non-TB    2022        4 69.6 0.0822
## 19   20-24     TB    2022        5 69.6 0.0822
## 20   20-24     TB    2018       12 69.6 0.0822
## 21   20-24     TB    2019        8 69.6 0.0822
## 22   20-24     TB    2021       16 69.6 0.0822
## 23   25-29 Non-TB    2017        5 64.6 0.0793
## 24   25-29 Non-TB    2018        6 64.6 0.0793
## 25   25-29     TB    2018       20 64.6 0.0793
## 26   25-29     TB    2019       10 64.6 0.0793
## 27   25-29 Non-TB    2020        2 64.6 0.0793
## 28   25-29     TB    2020       11 64.6 0.0793
## 29   25-29 Non-TB    2019        3 64.6 0.0793
## 30   25-29 Non-TB    2021        1 64.6 0.0793
## 31   25-29     TB    2021       16 64.6 0.0793
## 32   25-29     TB    2017       15 64.6 0.0793
## 33   25-29 Non-TB    2022        1 64.6 0.0793
## 34   25-29     TB    2022        9 64.6 0.0793
## 35   30-34 Non-TB    2017       12 59.6 0.0761
## 36   30-34 Non-TB    2022        4 59.6 0.0761
## 37   30-34 Non-TB    2018        3 59.6 0.0761
## 38   30-34     TB    2018       18 59.6 0.0761
## 39   30-34     TB    2020       20 59.6 0.0761
## 40   30-34 Non-TB    2019        4 59.6 0.0761
## 41   30-34     TB    2019       22 59.6 0.0761
## 42   30-34 Non-TB    2020        6 59.6 0.0761
## 43   30-34     TB    2017       17 59.6 0.0761
## 44   30-34 Non-TB    2021        4 59.6 0.0761
## 45   30-34     TB    2021       26 59.6 0.0761
## 46   30-34     TB    2022       19 59.6 0.0761
## 47   35-39 Non-TB    2020        8 54.7 0.0715
## 48   35-39 Non-TB    2018       14 54.7 0.0715
## 49   35-39 Non-TB    2017       25 54.7 0.0715
## 50   35-39     TB    2017       36 54.7 0.0715
## 51   35-39 Non-TB    2022        6 54.7 0.0715
## 52   35-39     TB    2022       27 54.7 0.0715
## 53   35-39     TB    2019       39 54.7 0.0715
## 54   35-39     TB    2018       41 54.7 0.0715
## 55   35-39 Non-TB    2019       11 54.7 0.0715
## 56   35-39     TB    2021       43 54.7 0.0715
## 57   35-39     TB    2020       41 54.7 0.0715
## 58   35-39 Non-TB    2021       13 54.7 0.0715
## 59   40-44 Non-TB    2018       26 49.7 0.0659
## 60   40-44 Non-TB    2020       16 49.7 0.0659
## 61   40-44 Non-TB    2022       14 49.7 0.0659
## 62   40-44 Non-TB    2021       25 49.7 0.0659
## 63   40-44     TB    2021       58 49.7 0.0659
## 64   40-44 Non-TB    2017       40 49.7 0.0659
## 65   40-44     TB    2017       57 49.7 0.0659
## 66   40-44     TB    2020       52 49.7 0.0659
```

```
## 67   40-44     TB    2022       49 49.7 0.0659
## 68   40-44     TB    2018       48 49.7 0.0659
## 69   40-44 Non-TB    2019       42 49.7 0.0659
## 70   40-44     TB    2019       52 49.7 0.0659
## 71   45-49     TB    2017       92 44.8 0.0604
## 72   45-49 Non-TB    2017       47 44.8 0.0604
## 73   45-49     TB    2018       77 44.8 0.0604
## 74   45-49 Non-TB    2018       53 44.8 0.0604
## 75   45-49 Non-TB    2020       39 44.8 0.0604
## 76   45-49     TB    2020       72 44.8 0.0604
## 77   45-49 Non-TB    2021       31 44.8 0.0604
## 78   45-49     TB    2021      106 44.8 0.0604
## 79   45-49     TB    2019       86 44.8 0.0604
## 80   45-49 Non-TB    2022       16 44.8 0.0604
## 81   45-49     TB    2022       59 44.8 0.0604
## 82   45-49 Non-TB    2019       46 44.8 0.0604
## 83   50-54 Non-TB    2020       51 39.9 0.0537
## 84   50-54 Non-TB    2017       85 39.9 0.0537
## 85   50-54 Non-TB    2021       51 39.9 0.0537
## 86   50-54 Non-TB    2018       69 39.9 0.0537
## 87   50-54     TB    2018       98 39.9 0.0537
## 88   50-54 Non-TB    2022       22 39.9 0.0537
## 89   50-54 Non-TB    2019       64 39.9 0.0537
## 90   50-54     TB    2019      101 39.9 0.0537
## 91   50-54     TB    2020       93 39.9 0.0537
## 92   50-54     TB    2021       93 39.9 0.0537
## 93   50-54     TB    2017      105 39.9 0.0537
## 94   50-54     TB    2022       91 39.9 0.0537
## 95   55-59 Non-TB    2020       70 35.1 0.0455
## 96   55-59     TB    2020      120 35.1 0.0455
## 97   55-59 Non-TB    2017       94 35.1 0.0455
## 98   55-59     TB    2017      126 35.1 0.0455
## 99   55-59 Non-TB    2022       37 35.1 0.0455
## 100  55-59 Non-TB    2018       75 35.1 0.0455
## 101  55-59     TB    2018       97 35.1 0.0455
## 102  55-59 Non-TB    2019       70 35.1 0.0455
## 103  55-59     TB    2019      129 35.1 0.0455
## 104  55-59 Non-TB    2021       51 35.1 0.0455
## 105  55-59     TB    2021      125 35.1 0.0455
## 106  55-59     TB    2022      102 35.1 0.0455
## 107  60-64 Non-TB    2018       80 30.2 0.0372
## 108  60-64     TB    2018      108 30.2 0.0372
## 109  60-64 Non-TB    2019       75 30.2 0.0372
## 110  60-64 Non-TB    2020       87 30.2 0.0372
## 111  60-64 Non-TB    2021       68 30.2 0.0372
## 112  60-64 Non-TB    2017      102 30.2 0.0372
## 113  60-64     TB    2017      139 30.2 0.0372
## 114  60-64 Non-TB    2022       37 30.2 0.0372
## 115  60-64     TB    2022       94 30.2 0.0372
## 116  60-64     TB    2021      144 30.2 0.0372
## 117  60-64     TB    2019      132 30.2 0.0372
## 118  60-64     TB    2020      140 30.2 0.0372
## 119  65-69     TB    2018      137 25.5 0.0296
## 120  65-69 Non-TB    2022       45 25.5 0.0296
## 121  65-69     TB    2022       93 25.5 0.0296
## 122  65-69 Non-TB    2018      103 25.5 0.0296
## 123  65-69 Non-TB    2020       61 25.5 0.0296
## 124  65-69     TB    2017      141 25.5 0.0296
## 125  65-69 Non-TB    2021       63 25.5 0.0296
## 126  65-69     TB    2021      152 25.5 0.0296
## 127  65-69 Non-TB    2017      102 25.5 0.0296
## 128  65-69     TB    2019      147 25.5 0.0296
## 129  65-69     TB    2020      124 25.5 0.0296
## 130  65-69 Non-TB    2019       78 25.5 0.0296
## 131  70-74 Non-TB    2017      102 20.8 0.0221
## 132  70-74 Non-TB    2018       90 20.8 0.0221
## 133  70-74     TB    2022       86 20.8 0.0221
```

```
## 134  70-74     TB    2017       94 20.8 0.0221
## 135  70-74 Non-TB    2020       77 20.8 0.0221
## 136  70-74 Non-TB    2022       31 20.8 0.0221
## 137  70-74 Non-TB    2019       76 20.8 0.0221
## 138  70-74     TB    2018      119 20.8 0.0221
## 139  70-74     TB    2019      122 20.8 0.0221
## 140  70-74     TB    2020      106 20.8 0.0221
## 141  70-74 Non-TB    2021       65 20.8 0.0221
## 142  70-74     TB    2021      105 20.8 0.0221
## 143  75-79 Non-TB    2017       74 16.4 0.0152
## 144  75-79     TB    2021       90 16.4 0.0152
## 145  75-79     TB    2017      105 16.4 0.0152
## 146  75-79 Non-TB    2020       51 16.4 0.0152
## 147  75-79 Non-TB    2021       43 16.4 0.0152
## 148  75-79 Non-TB    2018       82 16.4 0.0152
## 149  75-79     TB    2018      112 16.4 0.0152
## 150  75-79 Non-TB    2022       24 16.4 0.0152
## 151  75-79 Non-TB    2019       64 16.4 0.0152
## 152  75-79     TB    2019      112 16.4 0.0152
## 153  75-79     TB    2020      108 16.4 0.0152
## 154  75-79     TB    2022       72 16.4 0.0152
## 155  80-84 Non-TB    2017       52 12.5 0.0091
## 156  80-84 Non-TB    2018       50 12.5 0.0091
## 157  80-84 Non-TB    2020       25 12.5 0.0091
## 158  80-84 Non-TB    2021       34 12.5 0.0091
## 159  80-84     TB    2021       51 12.5 0.0091
## 160  80-84     TB    2017       76 12.5 0.0091
## 161  80-84     TB    2020       79 12.5 0.0091
## 162  80-84 Non-TB    2022       11 12.5 0.0091
## 163  80-84     TB    2022       41 12.5 0.0091
## 164  80-84     TB    2018       80 12.5 0.0091
## 165  80-84 Non-TB    2019       67 12.5 0.0091
## 166  80-84     TB    2019       75 12.5 0.0091
## 167    85+ Non-TB    2018       50  7.6 0.0063
## 168    85+     TB    2018       53  7.6 0.0063
## 169    85+ Non-TB    2019       26  7.6 0.0063
## 170    85+     TB    2019       42  7.6 0.0063
## 171    85+ Non-TB    2020       23  7.6 0.0063
## 172    85+     TB    2020       40  7.6 0.0063
## 173    85+ Non-TB    2021       28  7.6 0.0063
## 174    85+     TB    2021       29  7.6 0.0063
## 175    85+ Non-TB    2017       46  7.6 0.0063
## 176    85+     TB    2017       62  7.6 0.0063
## 177    85+ Non-TB    2022       14  7.6 0.0063
## 178    85+     TB    2022       16  7.6 0.0063
```

```
#Age standardized death rate #YLL
death.data %>% mutate(asdr=stdpop*l.discht) -> death.data
death.data %>% mutate(YLL= SLE*l.discht) ->death.data
death.data %>% mutate(YLL.100000=YLL/(stdpop*100000)) ->death.data


yll.all<-aggregate(cbind(l.discht,asdr,YLL,YLL.100000)~dx.date+group,death.data,sum)

yll.all %>% mutate(across(where(is.numeric), round, 1)) -> yll.all
```

```
## Warning: There was 1 warning in `mutate()`.
## ℹ In argument: `across(where(is.numeric), round, 1)`.
## Caused by warning:
## ! The `...` argument of `across()` is deprecated as of dplyr 1.1.0.
## Supply arguments directly to `.fns` through an anonymous function instead.
##
##   # Previously
##   across(a:b, mean, na.rm = TRUE)
##
##   # Now
##   across(a:b, \(x) mean(x, na.rm = TRUE))
```

```
yll.all %>% dplyr::rename(death=l.discht) -> yll.all
yll.all
```

```
##    dx.date  group death asdr   YLL YLL.100000
## 1     2017 Non-TB   793 29.0 23717        7.1
## 2     2018 Non-TB   714 24.8 20581        6.5
## 3     2019 Non-TB   636 22.9 18852        5.8
## 4     2020 Non-TB   522 18.9 15517        4.6
## 5     2021 Non-TB   479 17.0 13977        4.3
## 6     2022 Non-TB   267  9.9  8122        2.4
## 7     2017     TB  1081 41.2 33575        9.7
## 8     2018     TB  1027 38.4 31589        9.3
## 9     2019     TB  1086 41.2 33685        9.7
## 10    2020     TB  1040 40.2 33002        9.3
## 11    2021     TB  1059 43.2 34900        9.2
## 12    2022     TB   770 31.1 25168        6.7
```

```
#write.csv(yll.all,file="D:\\Plos one revise\\yll.all.csv")
```

```
cc.match2$strno <-nchar(cc.match2$l.pdx)

cc.match2 %>% filter(strno<=3) ->match.all0
cc.match2 %>% filter(strno==4) ->match.all1
cc.match2 %>% filter(strno>4) ->match.all2

match.all1$l.pdx<-substr(match.all1$l.pdx,1,nchar(match.all1$l.pdx)-1)
match.all2$l.pdx<-substr(match.all2$l.pdx,1,nchar(match.all2$l.pdx)-2)

deathcause<-rbind(match.all1,match.all2,match.all0)

deathcause %>% dplyr::rename("three_digit"="l.pdx")-> deathcause

icd10 <- icd10 %>% distinct(three_digit,.keep_all = TRUE)

deathcause<-merge(deathcause,icd10,by="three_digit")

deathcause<-deathcause %>% filter(l.discht==1)
deathcause$chapter[deathcause$chapter=="Congenital malformations, deformations and chromosomal abnormalities"]<-"Others"
deathcause$chapter[deathcause$chapter=="Diseases of the skin and subcutaneous tissue"]<-"Others"
deathcause$chapter[deathcause$chapter=="Factors influencing health status and contact with health services"]<-"Others"
deathcause$chapter[deathcause$chapter=="Symptoms, signs and abnormal clinical and laboratory findings, not elsewhere classif
ied"]<-"Others"


table1::table1(~chapter|group,data=deathcause)
```

	Non-TB (N=3408)	TB (N=6061)	Overall (N=9469)
**chapter**			
Certain conditions originating in the perinatal period	4 (0.1%)	2 (0.0%)	6 (0.1%)

	Non-TB (N=3408)	TB (N=6061)	Overall (N=9469)
Certain infectious and parasitic diseases	205 (6.0%)	4396 (72.5%)	4601 (48.6%)
Diseases of the blood and blood-forming organs and certain disorders involving the immune mechanism	23 (0.7%)	8 (0.1%)	31 (0.3%)
Diseases of the circulatory system	639 (18.8%)	250 (4.1%)	889 (9.4%)
Diseases of the digestive system	421 (12.4%)	155 (2.6%)	576 (6.1%)
Diseases of the eye and adnexa	1 (0.0%)	0 (0%)	1 (0.0%)
Diseases of the genitourinary system	290 (8.5%)	128 (2.1%)	418 (4.4%)
Diseases of the musculoskeletal system and connective tissue	78 (2.3%)	23 (0.4%)	101 (1.1%)
Diseases of the nervous system	69 (2.0%)	37 (0.6%)	106 (1.1%)
Diseases of the respiratory system	885 (26.0%)	693 (11.4%)	1578 (16.7%)
Endocrine, nutritional and metabolic diseases	63 (1.8%)	30 (0.5%)	93 (1.0%)
Injury, poisoning and certain other consequences of external causes	297 (8.7%)	68 (1.1%)	365 (3.9%)
Mental, Behavioral and Neurodevelopmental disorders	7 (0.2%)	5 (0.1%)	12 (0.1%)
Neoplasms	239 (7.0%)	155 (2.6%)	394 (4.2%)
Others	187 (5.5%)	111 (1.8%)	298 (3.1%)

```
head(deathcause)
```

```
##   three_digit      pid  tran_id    sex marry_status age_y    dx.date dx.dscdate
## 1         A02 49334861 70040169 Female            2    47 2019-02-05 2019-02-15
## 2         A02 53871711 39552441   Male            2    88 2017-05-17 2017-05-24
## 3         A02  1008526  3795437   Male            1    41 2017-10-03 2017-10-09
## 4         A04 37026340 82830570 Female            2    64 2017-04-11 2017-04-26
## 5         A04 36807781  7301511 Female            9    69 2017-05-19 2017-05-24
## 6         A09 19074296 49412692   Male            1    64 2019-02-28 2019-03-10
##   timeadm timedsc los dischs discht drg_nhso rw_nhso adjrw_nhso weight mth
## 1    1131    1012   0      2      1    18501    0.81       0.81     44   2
## 2    1051    1330   0      2      1     6103    1.53       1.53      0   5
## 3     922    1200   0      2      1     8530    1.08       1.08     50  10
## 4    1755    1130   0      2      1     4523    1.49       1.49      3   4
## 5     125    1149   0      9      8     6574    1.24       1.24      0   5
## 6     753    2310   0      3      4    10504    2.19       2.19     45   2
##   g_year   pdx  sdx1 sdx2 sdx3  sdx4 sdx5 sdx6 sdx7 sdx8 sdx9 sdx10 sdx11 sdx12
## 1   2562  A021 B3709 E876                                                      
## 2   2560  K409   C20 E119 E789   I10                                           
## 3   2561 M8696                                                                 
## 4   2560  A162  E871 E876  I10   K30 K922 M059                                 
## 5   2560  A049  E119 E782 I429 M0594 N179 R572  D65  I48 R570                  
## 6   2562  E110  A099 E871 E876  J960 R572                                      
##   sdx13 sdx14 sdx15 sdx16 sdx17 sdx18 sdx19 sdx20   proc1   proc2   proc3
## 1                                                                        
## 2                                                    1712    8876        
## 3                                                 8628+11                
## 4                                                    5794    9311    9312
## 5                                                 3895+11 3995+11 8801+11
## 6                                                    9604                
##     proc4   proc5   proc6   proc7  proc8   proc9  proc10  proc11 proc12 proc13
## 1                                                                              
## 2                                                                              
## 3                                                                              
## 4    9318    9335    9357    9383   9389    9396                               
## 5 8872+11 8961+11 8962+11 9604+11 966+11 9671+11 9904+11 9906+11               
## 6                                                                              
##   proc14 proc15 proc16 proc17 proc18 proc19 proc20 proc21            death_date
## 1                                                         2019-07-08 00:00:00.0
## 2                                                                              
## 3                                                                              
## 4                                                         2017-08-11 00:00:00.0
## 5                                                         2017-05-23 00:00:00.0
## 6                                                         2019-03-11 00:00:00.0
##   health_region superdistrict
## 1             1           217
## 2             6            72
## 3             7           184
## 4            10           125
## 5            13            24
## 6             1           219
##                                                                matchdup agegrp year
## 1  Female 47 2019-02-05 20190215 1131 1012 0 2 1 A021 0.8082 1 217  45-49 2019
## 2     Male 88 2017-05-17 20170524 1051 1330 0 2 1 K409 1.5326 6 72  85-89 2017
## 3    Male 41 2017-10-03 20171009 922 1200 0 2 1 M8696 1.0811 7 184  40-44 2017
## 4 Female 64 2017-04-11 20170426 1755 1130 0 2 1 A162 1.4948 10 125  60-64 2017
## 5   Female 69 2017-05-19 20170524 125 1149 0 9 8 A049 1.2392 13 24  65-69 2017
## 6      Male 64 2019-02-28 20190310 753 2310 0 3 4 E110 2.193 1 219  60-64 2019
##   month day       rwgroup                   matchid  group hfdate HF  ihddate
## 1     2   5 (0.545,0.875]  Female 45-49 2019 2 5 217 Non-TB     NA NO       NA
## 2     5  17  (1.49,1e+03]    Male 85-89 2017 5 17 72 Non-TB     NA NO 20170517
## 3    10   3  (0.875,1.49]   Male 40-44 2017 10 3 184 Non-TB     NA NO       NA
## 4     4  11  (0.875,1.49] Female 60-64 2017 4 11 125     TB     NA NO       NA
## 5     5  19  (0.875,1.49]  Female 65-69 2017 5 19 24 Non-TB     NA NO 20170519
## 6     2  28  (1.49,1e+03]   Male 60-64 2019 2 28 219 Non-TB     NA NO 20190228
##   IHD hivdate HIV   dmdate DM   cadate CA copddate COPD l.age_y  l.datedsc
## 1  NO      NA  NO       NA NO       NA NO       NA   NO      47 2019-07-08
## 2 IHD      NA  NO 20170517 DM 20170517 CA       NA   NO      93 2022-04-06
## 3  NO      NA  NO       NA NO       NA NO       NA   NO      35 2019-09-30
```

```
## 4  NO      NA  NO       NA NO       NA NO       NA   NO      64 2017-08-12
## 5 IHD      NA  NO 20170519 DM       NA NO       NA   NO      69 2017-05-24
## 6 IHD      NA  NO 20190228 DM       NA NO       NA   NO      64 2019-03-11
##   l.gyear l.discht distance weights subclass duration strno  X code
## 1    2562        1    0.329       1  2_34161      143     4 18  A02
## 2    2565        1    0.071       1  2_12426     1778     4 18  A02
## 3    2562        1    0.329       1  2_44986      721     4 18  A02
## 4    2560        1    0.307       1  2_22011      108     4 38  A04
## 5    2560        1    0.232       1  2_14069        0     4 38  A04
## 6    2562        1    0.231       1   2_9097        1     4 94  A09
##                                          short_desc
## 1                          Other salmonella infections
## 2                          Other salmonella infections
## 3                          Other salmonella infections
## 4                Other bacterial intestinal infections
## 5                Other bacterial intestinal infections
## 6 Infectious gastroenteritis and colitis, unspecified
##                                              major
## 1                          Other salmonella infections
## 2                          Other salmonella infections
## 3                          Other salmonella infections
## 4                Other bacterial intestinal infections
## 5                Other bacterial intestinal infections
## 6 Infectious gastroenteritis and colitis, unspecified
##                    sub_chapter                                  chapter
## 1 Intestinal Infectious Diseases Certain infectious and parasitic diseases
## 2 Intestinal Infectious Diseases Certain infectious and parasitic diseases
## 3 Intestinal Infectious Diseases Certain infectious and parasitic diseases
## 4 Intestinal Infectious Diseases Certain infectious and parasitic diseases
## 5 Intestinal Infectious Diseases Certain infectious and parasitic diseases
## 6 Intestinal Infectious Diseases Certain infectious and parasitic diseases
```

```
deathcause$inf <- ifelse(deathcause$chapter == "Certain infectious and parasitic diseases", 1, 0)
chisq.test(deathcause$inf, deathcause$group)
```

```
##
##  Pearson's Chi-squared test with Yates' continuity correction
##
## data:  deathcause$inf and deathcause$group
## X-squared = 3861, df = 1, p-value <0.0000000000000002
```

```
deathcause$resp <- ifelse(deathcause$chapter == "Diseases of the respiratory system", 1, 0)
chisq.test(deathcause$resp, deathcause$group)
```

```
##
##  Pearson's Chi-squared test with Yates' continuity correction
##
## data:  deathcause$resp and deathcause$group
## X-squared = 331, df = 1, p-value <0.0000000000000002
```

```
deathcause$circu <- ifelse(deathcause$chapter == "Diseases of the circulatory system", 1, 0)
chisq.test(deathcause$circu, deathcause$group)
```

```
##
##  Pearson's Chi-squared test with Yates' continuity correction
##
## data:  deathcause$circu and deathcause$group
## X-squared = 547, df = 1, p-value <0.0000000000000002
```

```
deathcause$dig <- ifelse(deathcause$chapter == "Diseases of the digestive system", 1, 0)
chisq.test(deathcause$dig, deathcause$group)
```

```
##
##  Pearson's Chi-squared test with Yates' continuity correction
##
## data:  deathcause$dig and deathcause$group
## X-squared = 365, df = 1, p-value <0.0000000000000002
```

```
deathcause$geni <- ifelse(deathcause$chapter == "Diseases of the genitourinary system", 1, 0)
chisq.test(deathcause$geni, deathcause$group)
```

```
##
##  Pearson's Chi-squared test with Yates' continuity correction
##
## data:  deathcause$geni and deathcause$group
## X-squared = 210, df = 1, p-value <0.0000000000000002
```

```
deathcause$neop <- ifelse(deathcause$chapter == "Neoplasms", 1, 0)
chisq.test(deathcause$neop, deathcause$group)
```

```
##
##  Pearson's Chi-squared test with Yates' continuity correction
##
## data:  deathcause$neop and deathcause$group
## X-squared = 107, df = 1, p-value <0.0000000000000002
```

```
deathcause$inj <- ifelse(deathcause$chapter == "Injury, poisoning and certain other consequences of external causes", 1, 0)
chisq.test(deathcause$inj, deathcause$group)
```

```
##
##  Pearson's Chi-squared test with Yates' continuity correction
##
## data:  deathcause$inj and deathcause$group
## X-squared = 337, df = 1, p-value <0.0000000000000002
```

```
deathcause$Others <- ifelse(deathcause$chapter == "Others", 1, 0)
chisq.test(deathcause$Others, deathcause$group)
```

```
##
##  Pearson's Chi-squared test with Yates' continuity correction
##
## data:  deathcause$Others and deathcause$group
## X-squared = 94, df = 1, p-value <0.0000000000000002
```

```
deathcause$musc <- ifelse(deathcause$chapter == "Diseases of the musculoskeletal system and connective tissue", 1, 0)
chisq.test(deathcause$musc, deathcause$group)
```

```
##
##  Pearson's Chi-squared test with Yates' continuity correction
##
## data:  deathcause$musc and deathcause$group
## X-squared = 74, df = 1, p-value <0.0000000000000002
```

```
deathcause$endo <- ifelse(deathcause$chapter == "Endocrine, nutritional and metabolic diseases", 1, 0)
chisq.test(deathcause$endo, deathcause$group)
```

```
##
##  Pearson's Chi-squared test with Yates' continuity correction
##
## data:  deathcause$endo and deathcause$group
## X-squared = 40, df = 1, p-value = 0.0000000003
```

```
deathcause$nerv <- ifelse(deathcause$chapter == "Diseases of the nervous system", 1, 0)
chisq.test(deathcause$nerv, deathcause$group)
```

```
##
##  Pearson's Chi-squared test with Yates' continuity correction
##
## data:  deathcause$nerv and deathcause$group
## X-squared = 38, df = 1, p-value = 0.0000000007
```

```
deathcause$bld <- ifelse(deathcause$chapter == "Diseases of the blood and blood-forming organs and certain disorders involvi
ng the immune mechanism", 1, 0)
chisq.test(deathcause$bld, deathcause$group)
```

```
##
##  Pearson's Chi-squared test with Yates' continuity correction
##
## data:  deathcause$bld and deathcause$group
## X-squared = 18, df = 1, p-value = 0.00002
```

```
deathcause$enta <- ifelse(deathcause$chapter == "Mental, Behavioral and Neurodevelopmental disorders", 1, 0)
chisq.test(deathcause$enta, deathcause$group)
```

```
## Warning in chisq.test(deathcause$enta, deathcause$group): Chi-squared
## approximation may be incorrect
```

```
##
##  Pearson's Chi-squared test with Yates' continuity correction
##
## data:  deathcause$enta and deathcause$group
## X-squared = 2, df = 1, p-value = 0.2
```

```
deathcause$perinatal <- ifelse(deathcause$chapter == "Certain conditions originating in the perinatal period", 1, 0)
fisher.test(deathcause$perinatal, deathcause$group)
```

```
##
##  Fisher's Exact Test for Count Data
##
## data:  deathcause$perinatal and deathcause$group
## p-value = 0.2
## alternative hypothesis: true odds ratio is not equal to 1
## 95 percent confidence interval:
##  0.025 1.961
## sample estimates:
## odds ratio
##       0.28
```

```
deathcause$eye <- ifelse(deathcause$chapter == "Diseases of the eye and adnexa", 1, 0)
chisq.test(deathcause$eye, deathcause$group)
```

```
## Warning in chisq.test(deathcause$eye, deathcause$group): Chi-squared
## approximation may be incorrect
```

```
##
##  Pearson's Chi-squared test with Yates' continuity correction
##
## data:  deathcause$eye and deathcause$group
## X-squared = 0.09, df = 1, p-value = 0.8
```

```
pvals <- c(0.00001,0.00001,0.00001,0.000001,0.00001,0.00001,0.000001,0.000001,0.000001,0.000006,0.00009,0.002,0.5,0.7,0.9)

# Bonferroni adjustment
pvals_bonf <- p.adjust(pvals, method = "bonferroni")
pvals_bonf
```

```
##  [1] 0.000150 0.000150 0.000150 0.000015 0.000150 0.000150 0.000015 0.000015
##  [9] 0.000015 0.000090 0.001350 0.030000 1.000000 1.000000 1.000000
```

#Figure 2 Cumulative incidence rate per 100,000 person-years of cardiopulmonary sequelae and death of TB compared to non-TB group

```
#dfList[[79]](ICD-10 I32 (Pericarditis) in TB and non TB), n=117944
#dfList[[20]] (ICD-10 J93 (Pneumothorax) in TB and non TB), n=115654
#dfList[[50]] (ICD-10 J85 (Abscess of lung and mediastinum) in TB and non TB),n=117484
#dfList[[27]] (ICD-10 J47 (Bronchiectasis) in TB and non TB),n=116664
#dfList[[46]] (ICD-10 J86 (Pyothorax) in TB and non TB), n= 117458
#dfList[[11]] (ICD-10 J90 (Pleural effusion) in TB and non TB), n=112118
#dfList[[4]] (ICD-10 J15 (Bacterial pneumonia) in TB and non TB),n=104620
#dfList[[2]] (ICD-10 J96 (Respiratory failure) in TB and non TB), n=97304

mdata.all<-dfList[[2]]
table(mdata.all$pdx.2)
```

```
##
##    J15     No
##   2681 101939
```

```
mdata.all$event<-mdata.all$pdx.2
table(mdata.all$event,mdata.all$group)
```

```
##
##       Non-TB    TB
##   J15   1168  1513
##   No   51142 50797
```

```
mdata.all$event<-factor(mdata.all$event)
mdata.all$event<-as.numeric(mdata.all$event)
mdata.all$event<-ifelse(mdata.all$event==2,0,1)
#mdata.all$event<-factor(mdata.all$event)
mdata.all$group<-factor(mdata.all$group)

mdata.all$group<-fct_rev(mdata.all$group)
options(scipen=999)
mdata.all$duration<-as.numeric(mdata.all$duration)

# Risk table from survival distribution
fit2<- survfit(Surv(duration, event) ~ group, data = mdata.all)
tables <-ggsurvtable(fit2, data = mdata.all,
  y.text = FALSE,ylab="",xlab="Days since discharge",legend=FALSE,break.time.by = 365)
```

```
## Warning: ggtheme is not a valid theme.
## Please use `theme()` to construct themes.
```

```
## Warning: tables.theme is not a valid theme.
## Please use `theme()` to construct themes.
```

```
## Ignoring unknown labels:
## • colour : "Strata"
```

```
## Warning: ggtheme is not a valid theme.
## Please use `theme()` to construct themes.
## tables.theme is not a valid theme.
## Please use `theme()` to construct themes.
```

```
## Ignoring unknown labels:
## • colour : "Strata"
```

```
## Warning: ggtheme is not a valid theme.
## Please use `theme()` to construct themes.
## tables.theme is not a valid theme.
## Please use `theme()` to construct themes.
```

```
## Ignoring unknown labels:
## • colour : "Strata"
```

```
# Risk table
t4<-tables$cumevents+annotate("text", x=2, y=2.5, label= "TB")+annotate("text", x=100, y=1.5, label= "non-TB")

###############################

#Plot of cumulative incidence per 100 000 person-year
mdata.all %>% mutate(persony=duration/365)->mdata.all
mdata.all %>% group_by(group) %>% summarize(tpersony=sum(persony)) -> mdata.allpy
library(broom)
survfit(Surv(duration, event)~group, data=mdata.all) %>%
summary(fit, times = c(1, 10, 1*(1:2500))) -> sumfit
#In life table, incidence is calculated based on number at risk, not by person-years at risk
#We will calculate incidence rate per 100,000 person year from life table table

sumfit<-cbind(sumfit$time,sumfit$n.risk,sumfit$n.event,sumfit$strata)
colnames(sumfit)<-c("Time","n.risk","Event","Strata")
sumfit<-data.frame(sumfit)
sumfit %>% filter(Strata==2) -> sumfit2
sumfit %>% filter(Strata==1) -> sumfit1

#Person year at risk for non-TB
py<-mdata.allpy$tpersony[2]
#Cumulative event for non-TB
c.event<-cumsum(sumfit2$Event)
#time, cumulative event, person-year at risk table
sumfit2<-cbind(sumfit2,c.event,py)

#Person year at risk for TB
py<-mdata.allpy$tpersony[1]
#Cumulative event of TB
c.event<-cumsum(sumfit1$Event)
#time, cumulative event, person-year at risk table
sumfit1<-cbind(sumfit1,c.event,py)

#Merge time, cumulative event, person-year at risk of TB and non TB
sumfit.all<-rbind(sumfit1,sumfit2)
#Calculate Survival probability by person-year at risk
sumfit.all %>% mutate(st=(1-(c.event/py)))-> sumfit.all
#Calculate cumulative incidence rate by person-year at risk
sumfit.all %>% mutate(inc=1-st)-> sumfit.all
#Calculate cumulative incidence rate per 100,000 person-year at risk
sumfit.all %>% mutate(inc.100000=inc*100000) ->sumfit.all

#Plot cumulative incidence rate per 100,000 person-year at risk
sumfit.all$Strata<-factor(sumfit.all$Strata)
fig4<-ggplot(data=sumfit.all, aes(x=Time, y=inc.100000, group=Strata)) +
  geom_line(aes(linetype=Strata))+
  scale_x_continuous(breaks=seq(0,2000,by=365),name = "Days since discharge")+
  scale_y_continuous(name= "Cumulative incidence rate\nper 100000 person-years")+
  theme_bw()+ theme(legend.position="none")+theme(axis.text.x = element_text(size = 12),
        axis.text.y = element_text(size = 12),
        axis.title.x = element_text(size = 12),
        axis.title.y = element_text(size = 12)
        )+ labs(title = "7.Bacterial pneumonia (J15 ICD-10)")+
 #Change y values
   annotate("text", x=500, y=750, label= "TB")+
  annotate("text", x=500, y=500, label= "non-TB")

fig4
```

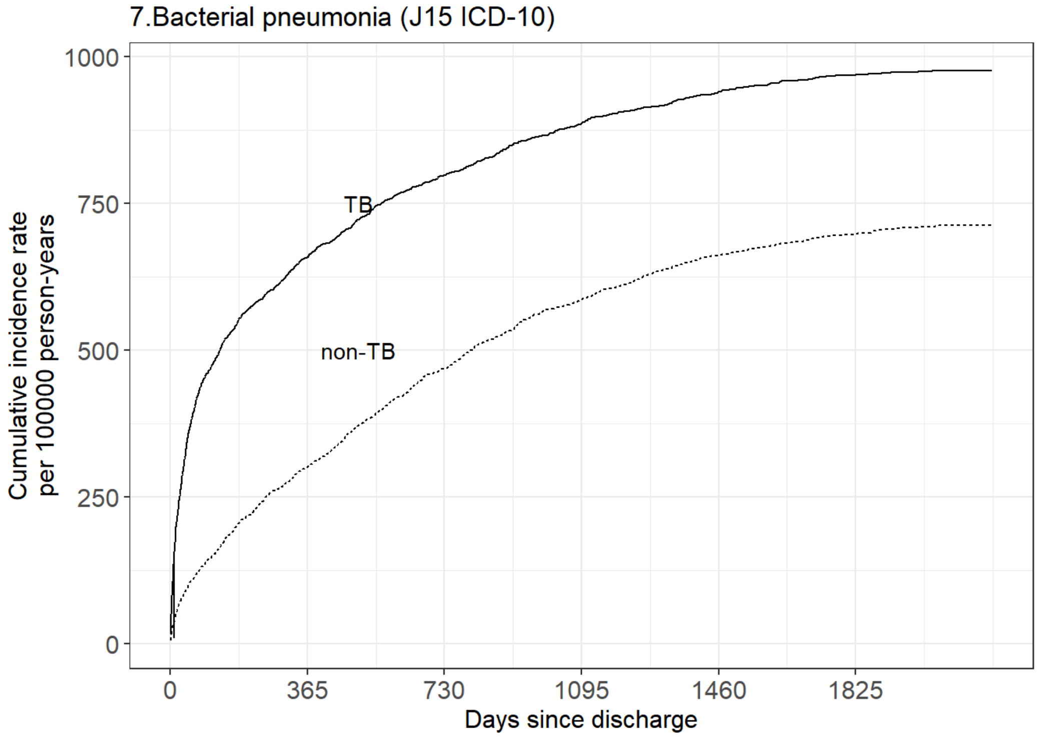


```
t4
```

```
## Ignoring unknown labels:
## • colour : "Strata"
```

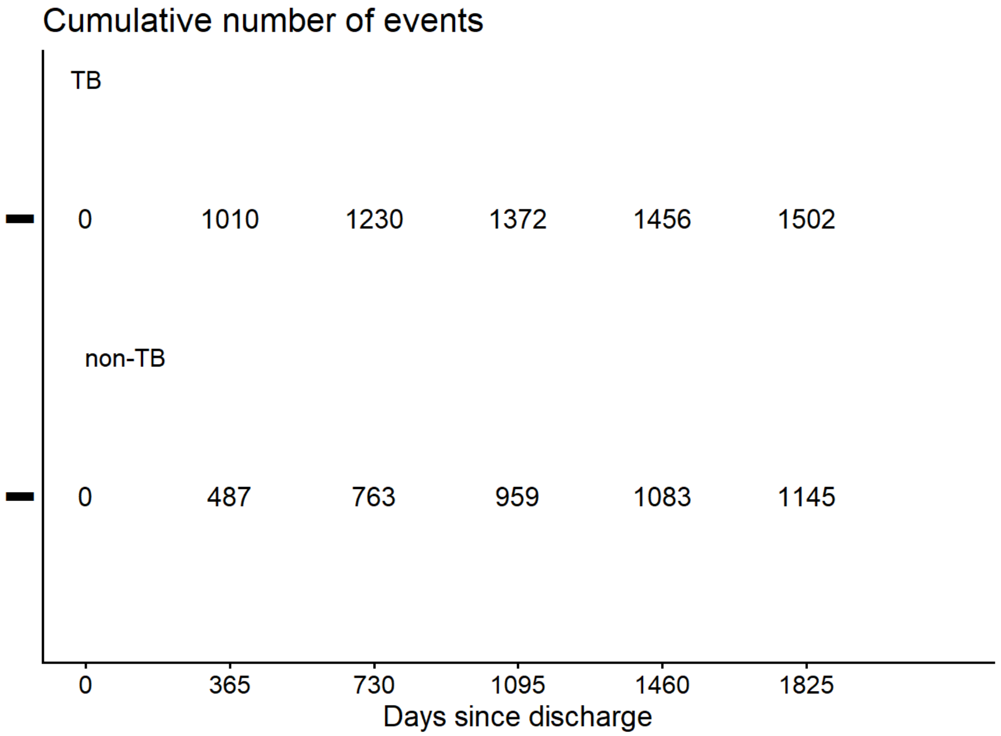


```
#setwd("D:\\Plos one revise\\Figure 3")
#tiff("Fig1-4.tiff", units="in", width=10, height=8.5, res=500)
#Combine risk table and cumulative incidence plot
#ggarrange(fig1,fig2, t1,t2, fig3,fig4,t3,t4,
  #                 ncol = 2,nrow = 4, widths = c(2, #2),heights=c(1.5,1.4))
#dev.off()
```

#Figure 2 Cumulative incidence rate per 100,000 person-years of death of TB compared to non-TB group

```
cc.match$event<-cc.match$l.discht
table(cc.match$event,cc.match$group)
```

```
##
##     Non-TB    TB
##   0  55616 52964
##   1   3411  6063
```

```
cc.match$event<-factor(cc.match$event)
cc.match$event<-as.numeric(cc.match$event)
cc.match$event<-ifelse(cc.match$event==2,1,0)
cc.match$group<-factor(cc.match$group)
cc.match$group<-relevel(cc.match$group,ref="TB")
class(cc.match$l.datedsc)
```

```
## [1] "Date"
```

```
cc.match$l.datedsc<-ymd(cc.match$l.datedsc)
cc.match$dx.date<-ymd(cc.match$dx.date)
cc.match %>% filter(l.discht==0) ->cc.match1
cc.match %>% filter(l.discht==1) ->cc.match0


cc.match1$l.datedsc <-"2022-12-30"
cc.match1$l.datedsc<-as.Date(cc.match1$l.datedsc)
cc.match<-rbind(cc.match1,cc.match0)


cc.match %>% mutate(duration=l.datedsc-dx.date) -> cc.match
cc.match$duration<-as.numeric(cc.match$duration)
cc.match$duration[cc.match$duration==0]<-1

fit2<- survfit(Surv(duration, event) ~ group, data = cc.match)


tables <- ggsurvtable(fit2, data = cc.match, xlimit=c(0,2500),
  y.text = FALSE,ylab="",xlab="Days since discharge",legend=FALSE,break.time.by = 365)
```

```
## Warning: ggtheme is not a valid theme.
## Please use `theme()` to construct themes.
```

```
## Warning: tables.theme is not a valid theme.
## Please use `theme()` to construct themes.
```

```
## Ignoring unknown labels:
## • colour : "Strata"
```

```
## Warning: ggtheme is not a valid theme.
## Please use `theme()` to construct themes.
## tables.theme is not a valid theme.
## Please use `theme()` to construct themes.
```

```
## Ignoring unknown labels:
## • colour : "Strata"
```

```
## Warning: ggtheme is not a valid theme.
## Please use `theme()` to construct themes.
## tables.theme is not a valid theme.
## Please use `theme()` to construct themes.
```

```
## Ignoring unknown labels:
## • colour : "Strata"
```

```
# Risk table
tD<-tables$cumevents+annotate("text", x=2, y=2.5, label= "TB")+annotate("text", x=100, y=1.5, label= "non-TB")

###############################

#Plot of cumulative incidence per 100000 person-year
cc.match %>% mutate(persony=duration/365)->cc.match
cc.match %>% group_by(group) %>% summarize(tpersony=sum(persony)) -> cc.matchpy
library(broom)
survfit(Surv(duration, event)~group, data=cc.match) %>%
summary(fit, times = c(1, 10, 1*(1:2500))) -> sumfit1
#Calculate incidence rate per 100,000 person year from life table table
#In life table, incidence is calculated based on number at risk, not by person-years at risk
sumfit<-cbind(sumfit1$time,sumfit1$n.risk,sumfit1$n.event,sumfit1$strata)
colnames(sumfit)<-c("Time","n.risk","Event","Strata")
sumfit<-data.frame(sumfit)
sumfit %>% filter(Strata==2) -> sumfit2

sumfit %>% filter(Strata==1) -> sumfit1

#Person year at risk for non-TB
py<-cc.matchpy$tpersony[2]
#Cumulative event for non-TB
c.event<-cumsum(sumfit2$Event)
#time, cumulative event, person-year at risk table
sumfit2<-cbind(sumfit2,c.event,py)

#Person year at risk for TB
py<-cc.matchpy$tpersony[1]
#Cumulative event of TB
c.event<-cumsum(sumfit1$Event)
#time, cumulative event, person-year at risk table
sumfit1<-cbind(sumfit1,c.event,py)

#Merge time, cumulative event, person-year at risk of TB and non TB
sumfit.all<-rbind(sumfit1,sumfit2)
#Calculate Survival probability by person-year at risk
sumfit.all %>% mutate(st=(1-(c.event/py)))-> sumfit.all
#Calculate cumulative incidence rate by person-year at risk
sumfit.all %>% mutate(inc=1-st)-> sumfit.all
#Calculate cumulative incidence rate per 100,000 person-year at risk
sumfit.all %>% mutate(inc.100000=inc*100000) ->sumfit.all

#Plot cumulative incidence rate per 100,000 person-year at risk
sumfit.all$Strata<-factor(sumfit.all$Strata)
figD<-ggplot(data=sumfit.all, aes(x=Time, y=inc.100000, group=Strata)) +
  geom_line(aes(linetype=Strata))+
  scale_x_continuous(breaks=seq(0,2000,by=365),name = "Days since discharge")+
  scale_y_continuous(name= "Cumulative incidence rate\nper 100000 person-years")+
  theme_bw()+ theme(legend.position="none")+theme(axis.text.x = element_text(size = 12),
        axis.text.y = element_text(size = 12),
        axis.title.x = element_text(size = 12),
        axis.title.y = element_text(size = 12))+labs(title = "Death")+
  annotate("text", x=500, y=3500, label= "TB")+
  annotate("text", x=500, y=1700, label= "non-TB")
```

#Death

```r
#setwd("D:\\Post TB\\TB data\\Results\\Figures\\Figures, revised")
#tiff("Death.tiff", units="in", width=6, height=4.6, res=500)
#Combine risk table and cumulative incidence plot
#ggarrange(fig,t1,
#                       ncol = 1, nrow = 2, widths = c(2, #1),heights=c(1.5,1.4))
#dev.off()
```

```r
library(powerSurvEpi)
```

```
## Warning: package 'powerSurvEpi' was built under R version 4.4.3
```

```r
powerCT.default0(k=1, m=28, RR=4.6, alpha = 0.05)
```

```
## [1] 0.93
```

```r
powerCT.default0(k=1, m=347, RR=3.3, alpha = 0.05)
```

```
## [1] 1
```

```r
powerCT.default0(k=1, m=120, RR=2.2, alpha = 0.05)
```

```
## [1] 0.98
```

```r
powerCT.default0(k=1, m=335, RR=3.1, alpha = 0.05)
```

```
## [1] 1
```

```r
powerCT.default0(k=1, m=159, RR=1.7, alpha = 0.05)
```

```
## [1] 0.9
```

```r
powerCT.default0(k=1, m=215, RR=1.5, alpha = 0.05)
```

```
## [1] 0.83
```

```r
powerCT.default0(k=1, m=2681, RR=1.3, alpha = 0.05)
```

```
## [1] 1
```

```r
powerCT.default0(k=1, m=4638, RR=1.1, alpha = 0.05)
```

```
## [1] 0.9
```

```r
powerCT.default0(k=1, m=278, RR=2.6, alpha = 0.05)
```

```
## [1] 1
```

```r
powerCT.default0(k=1, m=58, RR=18.3, alpha = 0.05)
```

```
## [1] 1
```

```
powerCT.default0(k=1, m=967, RR=2.0, alpha = 0.05)
```

```
## [1] 1
```
