# Supplementary material for: Readmission and survival of hospitalized pulmonary tuberculosis patients: a nationwide record-based cohort analysis in Thailand (2017–2022)
Source: Infect Dis Poverty. 2026 Jun 15;15:67. doi: 10.1186/s40249-026-01467-0 (PMC13267298; doi:10.1186/s40249-026-01467-0)
Supplement: Supplementary file 2 — Supplementary material 2. [file 40249_2026_1467_MOESM2_ESM.docx]

**Supplementary Table 1 Distribution of primary ICD–10 diagnoses of matched non–TB patients admitted for hospital**

| ICD–10 diagnoses of non–TB patients admitted for hospital | *n* (%) |
| --- | --- |
| Total | *N* = 59, 027 |
| Injury, poisoning and certain other consequences of external causes | 8763 (14.9) |
| Diseases of the digestive system | 7933 (13.5) |
| Diseases of the circulatory system | 6573 (11.2) |
| Diseases of the respiratory system | 5975 (10.1) |
| Diseases of the genitourinary system | 5045 (8.6) |
| Certain infectious and parasitic diseases | 3978 (6.7) |
| Symptoms, signs and abnormal clinical and laboratory findings, not  elsewhere classified | 3902 (6.6) |
| Diseases of the eye and adnexa | 3159 (5.4) |
| Diseases of the musculoskeletal system and connective tissue | 2285 (3.9) |
| Neoplasms | 2196 (3.7) |
| Mental, Behavioral and Neurodevelopmental disorders | 1973 (3.3) |
| Diseases of the skin and subcutaneous tissue | 1834 (3.1) |
| Endocrine, nutritional and metabolic diseases | 1803 (3.1) |
| Diseases of the nervous system | 1325 (2.2) |
| Diseases of the blood and blood-forming organs and certain disorders  involving the immune mechanism | 814 (1.4) |
| Factors influencing health status and contact with health services | 793 (1.3) |
| Pregnancy, childbirth and the puerperium | 417 (0.6) |
| Diseases of the ear and mastoid process | 186 (0.3) |
