# Supplementary material for: Readmission and survival of hospitalized pulmonary tuberculosis patients: a nationwide record-based cohort analysis in Thailand (2017–2022)
Source: Infect Dis Poverty. 2026 Jun 15;15:67. doi: 10.1186/s40249-026-01467-0 (PMC13267298; doi:10.1186/s40249-026-01467-0)
Supplement: Supplementary file 3 — Supplementary material 3. [file 40249_2026_1467_MOESM3_ESM.docx]

**Supplementary Table 2 Readmission incidence density per 10,000 person–year among TB and their matched non–TB**

| **ICD–10** | **Thoracic, and extra–thoracic disease events** | **Matched** | | **Unmatched** |
| --- | --- | --- | --- | --- |
|  |  | **non–TB** | **TB** | **TB** |
|  |  | **Incidence per 10,000 person –years** | **Incidence per 10,000 person –years** | **Incidence per 10,000 person –years** |
| Thoracic disease events | |  |  |  |
| Cardiovascular diseases | |  |  |  |
| I10 | Essential (primary) hypertension | 445.9  (436.3**–**455.7) | 168.4  (162.4**–**174.6) | 229.5  (223.2**–**235.8) |
| I48 | Atrial fibrillation and flutter | 58.5 (55.1**–**62.2) | 24.7 (22.4**–**27.1) | 32.1 (29.8**–**34.5) |
| I50 | Heart failure | 40.4 (37.5**–**43.4) | 21.6 (19.5**–**23.8) | 42.4 (39.8**–**45.2) |
| I25 | Chronic ischemic heart disease | 70.8 (67.0**–**74.7) | 19.9 (17.9**–**22.1) | 27.1 (24.9**–**29.3) |
| I69 | Sequelae of cerebrovascular disease | 69.0 (65.3**–**72.9) | 16.2 (14.4**–**18.2) | 21.0 (19.2**–**23.0) |
| I95 | Hypotension | 16.5 (14.7**–**18.4) | 15.4 (13.6**–**17.3) | 20.6 (18.8**–**22.6) |
| I21 | Acute myocardial infarction | 29.9 (27.4**–**32.5) | 10.3 (8.9**–**11.9) | 13.6 (12.1**–**15.3) |
| I63 | Cerebral infarction | 47.7 (44.6**–**51.0) | 10.1 (8.7**–**11.7) | 12.9 (11.4**–**14.5) |
| I47 | Paroxysmal tachycardia | 11.7 (10.2**–**13.4) | 8.3 (7.0**–**9.8) | 10.4 (9.1**–**11.8) |
| I32 | Pericarditis | 0.3 (0.1**–**0.6) | 1.6 (1.1–2.3) | 1.5 (1.1**–**2.1) |
| Pulmonary diseases | |  |  |  |
| J96 | Respiratory failure | 99.1 (94.6**–**103.8) | 111.9  (107.1**–**117.0) | 153.7  (148.6**–**158.9) |
| J15 | Bacterial pneumonia | 49.1 (45.9**–**52.4) | 81.8 (77.7**–**86.2) | 103.9 (99.7**–**108.2) |
| J90 | Pleural effusion | 19.0 (17.0**–**21.1) | 33.5 (30.9**–**36.3) | 37.5 (35.0**–**40.1) |
| J93 | Pneumothorax | 3.3 (2.6**–**4.3) | 18.9 (16.9**–**21.0) | 24.2 (22.2**–**26.4) |
| J47 | Bronchiectasis | 3.9 (3.0**–**4.9) | 15.4 (13.6**–**17.3) | 17.4 (15.7**–**19.2) |
| J12 | Viral pneumonia | 20.0 (18.0**–**22.2) | 8.4 (7.1**–**9.8) | 11.9 (10.5**–**13.5) |
| J45 | Asthma | 19.1 (17.1**–**21.2) | 6.7 (5.5**–**8.0) | 9.3 (8.1**–**10.7) |
| J20 | Acute bronchitis | 15.4 (13.7**–**17.3) | 6.3 (5.2**–**7.5) | 11.4 (10.0**–**12.8) |
| J86 | Pyothorax | 3.3 (2.6**–**4.3) | 6.1 (5.0**–**7.4) | 7.7 (6.6**–**8.9) |
| J69 | Pneumonitis due to solids and liquids | 13.3 (11.7**–**15.1) | 5.9 (4.8**–**7.2) | 6.1 (5.2**–**7.3) |
| J85 | Abscess of lung and mediastinum | 2.1 (1.5**–**2.9) | 4.8 (3.8**–**5.9) | 5.3 (4.4**–**6.3) |
| Extra–thoracic diseases | |  |  |  |
| E83 | Disorders of mineral metabolism | 129.7  (124.5**–**135.0) | 103.5  (98.8**–**108.4) | 128.6  (124.0**–**133.4) |
| N18 | Chronic kidney disease | 218.2 (211.5**–**225.1) | 84.4 (80.2**–**88.8) | 99.9 (95.8**–**104.1) |
| N17 | Acute kidney failure | 121.6 (116.6**–**126.8) | 79.2 (75.1**–**83.4) | 109.5 (105.2**–**113.9) |
| E78 | Disorders of lipoprotein metabolism and other lipidemias | 215.2 (208.6**–**222.1) | 60.3 (56.8**–**64.1) | 87.8 (83.9**–**91.8) |
| M10 | Arthropathy | 73.4 (69.5**–**77.4) | 42.9 (39.9**–**46.1) | 48.2 (45.3**–**51.2) |
| N39 | Other disorders of urinary system | 98.8 (94.3**–**103.4) | 38.8 (35.9**–**41.8) | 52.6 (49.6**–**55,7) |
| K92 | Other diseases of digestive system | 55.6 (52.2**–**59.1) | 34.4 (31.7**–**37.2) | 43.2 (40.5**–**46.0) |
| K75 | Inflammatory liver diseases | 13.8 (12.1**–**15.6) | 34.3 (31.6**–**37.2) | 45.3 (42.6**–**48.2) |
| E16 | Other disorders of pancreatic internal secretion | 29.7 (27.2**–**32.3) | 28.9 (26.4**–**31.5) | 40.0 (37.4**–**42.7) |
| K74 | Fibrosis and cirrhosis of liver | 50.2 (47.0**–**53.6) | 27.4 (25.0**–**30.0) | 30.1 (27.9**–**32.5) |
| K70 | Alcoholic liver disease | 39.7 (36.9**–**42.7) | 21.1 (19.0**–**23.4) | 21.4 (19.5**–**23.4) |
| E22 | Hyperfunction of pituitary gland | 3.6 (2.8**–**4.6) | 11.6 (10.0**–**13.3) | 14.2 (12.7**–**15.9) |

ICD–10, International Classification of Diseases, Tenth Revision; TB, Tuberculosis

Note: Supplementary Table 2 shows that significant readmission incidence between matched TB patients and non-TB controls using a stratified log-rank test, with statistical significance defined as *P* < 0.05.
