# Supplementary material for: Readmission and survival of hospitalized pulmonary tuberculosis patients: a nationwide record-based cohort analysis in Thailand (2017–2022)
Source: Infect Dis Poverty. 2026 Jun 15;15:67. doi: 10.1186/s40249-026-01467-0 (PMC13267298; doi:10.1186/s40249-026-01467-0)
Supplement: Supplementary file 4 — Supplementary material 4. [file 40249_2026_1467_MOESM4_ESM.docx]

**Supplementary Table 3 Post–hoc power analysis demonstrating sufficient power to detect difference in survival probability between matched TB and non-TB patients in Thailand (2017–2022)**

| **ICD–10** | **Thoracic and extra–thoracic diseases** |  | **Ratio of participants in TB compared to**  **Non–TB**  **(k)** | **Total number of diseases in both groups**  **(m)** | **Hazard ratio** | **Type I error rate**  **(alpha)** | **Power** |
| --- | --- | --- | --- | --- | --- | --- | --- |
| Thoracic diseases | |  |  |  |  |  |  |
| Cardiovascular diseases | |  |  |  |  |  |  |
| I32 | Pericarditis |  | 1 | 33 | 5.4 | 0.05 | 0.97 |
| Pulmonary diseases | |  |  |  |  |  |  |
| J93 | Pneumothorax |  | 1 | 395 | 5.4 | 0.05 | 1.00 |
| J47 | Bronchiectasis |  | 1 | 343 | 3.8 | 0.05 | 1.00 |
| J85 | Abscess of lung and mediastinum |  | 1 | 124 | 2.2 | 0.05 | 0.98 |
| J90 | Pleural effusion |  | 1 | 940 | 1.7 | 0.05 | 1.00 |
| J86 | Pyothorax |  | 1 | 169 | 1.7 | 0.05 | 0.92 |
| J15 | Bacterial pneumonia |  | 1 | 2344 | 1.6 | 0.05 | 1.00 |
| J96 | Respiratory failure |  | 1 | 3787 | 1.1 | 0.05 | 0.83 |
| Extra–thoracic diseases | |  |  |  |  |  |  |
| E22 | Hyperfunction of pituitary gland |  | 1 | 271 | 3.1 | 0.05 | 1.00 |
| K75 | Inflammatory liver diseases |  | 1 | 859 | 2.4 | 0.05 | 1.00 |

ICD–10, International Classification of Diseases, Tenth Revision; TB, Tuberculosis
