# Supplementary material for: Readmission and survival of hospitalized pulmonary tuberculosis patients: a nationwide record-based cohort analysis in Thailand (2017–2022)
Source: Infect Dis Poverty. 2026 Jun 15;15:67. doi: 10.1186/s40249-026-01467-0 (PMC13267298; doi:10.1186/s40249-026-01467-0)
Supplement: Supplementary file 5 — Supplementary material 5. [file 40249_2026_1467_MOESM5_ESM.docx]

**Supplementary Table 4 Incidence of hospital death per 10,000 person–year among TB and their matched non–TB**

| **ICD-10** | **Cause–specific death** | **Matched** | | **Unmatched** |
| --- | --- | --- | --- | --- |
|  |  | **Non**–**TB** | **TB** | **TB** |
| Total | | 186.9  (180.6–193.4) | 390.6  (381.1–400.3) | 369.3  (361.4–377.4) |
| A00–A99 | Certain infectious and parasitic diseases | 10.9  (9.4–12.6) | 334.1  (325.3–343.1) | 296.1  (289.0–303.3) |
| A15–A19 | - TB | 0 | 328.6  (319.9–337.5) | 283  (276.9–291.0) |
| J00–J99 | Diseases of the respiratory system | 44.2  (41.1–47.4) | 28.2  (25.7–30.9) | 37.9  (35.4–40.6) |
| I00–I99 | Diseases of the circulatory system | 37.1  (34.3–40.0) | 5.5  (4.4–6.7) | 9.1  (7.9–10.5) |
| K00-K99 | Diseases of the digestive system | 23.7  (21.5–26.1) | 5.0  (3.9–6.2) | 5.7  (4.7–6.7) |
| B00–C99 | Neoplasms | 15.5  (13.7–17.4) | 4.8  (3.8–6.0) | 6.1  (5.1–7.2) |
| Z00–Z99,  R00–R99 | Others | 10.0  (8.6–11.6) | 4.8  (3.8–6.0) | 4.0  (3.2–4.9) |
| N00–N99 | Diseases of the genitourinary system | 16.3  (14.5–18.3) | 3.9  (3.0–5.0) | 4.4  (3.6–5.4) |
| G00–G99 | Diseases of the nervous system | 3.6  (2.8–4.6) | 1.4  (0.9–2.1) | 1.4  (0.98–2.02) |
| E00–E90 | Endocrine, nutritional and metabolic diseases | 3.3  (2.5–4.3) | 1.3  (0.8–2.0) | 1.7  (1.2–2.3) |
| S00–T98 | Injury, poisoning and certain other consequences of external causes | 16.4  (14.6–18.4) | 0.7  (0.3–1.2) | 1.2  (0.7–1.7) |
| M00–M99 | Diseases of the musculoskeletal system and connective tissue | 4.0  (3.1–5.0) | 0.3  (0.1–0.7) | 0.44  (0.21–0.82) |
| D50–D89 | Diseases of the blood and blood-forming organs and certain disorders involving the immune mechanism | 0.9  (0.5–1.5) | 0.06  (0.001–0.34) | 0.3  (0.1–0.6) |
| F00–F99 | Mental, Behavioral and Neurodevelopmental disorders | 0.3  (0.1–0.7) | 0.06  (0.001–0.34) | 0.17  (0.04–0.45) |

ICD–10, International Classification of Diseases, Tenth Revision; TB, Tuberculosis

Note: Supplementary Table 4 shows that significant incidence of cause-specific deaths between matched TB patients and non-TB controls using a stratified log-rank test, with statistical significance defined as *P* < 0.05.
